# Pparg signaling controls bladder cancer subtype and immune exclusion

**DOI:** 10.1038/s41467-021-26421-6

**Published:** 2021-10-25

**Authors:** Tiffany Tate, Tina Xiang, Sarah E. Wobker, Mi Zhou, Xiao Chen, Hyunwoo Kim, Ekatherina Batourina, Chyuan-Sheng Lin, William Y. Kim, Chao Lu, James M. Mckiernan, Cathy Lee Mendelsohn

**Affiliations:** 1 Department of Urology, Columbia University Irving Medical Center, New York, NY 10032 USA; 2 Department of Pathology and Cell Biology, Columbia University Irving Medical Center, New York, NY 10032 USA; 3 Department of Genetics and Development, Columbia University Irving Medical Center, New York, NY 10032 USA; 4 Columbia Stem Cell Initiative, Columbia University Irving Medical Center, New York, NY 10032 USA; 5 Department of Pathology and Laboratory Medicine, University of North Carolina at Chapel Hill, Chapel Hill, NC 27599 USA; 6 Lineberger Comprehensive Cancer Center, University of North Carolina at Chapel Hill, Chapel Hill, NC 27599 USA; 7 Transgenic Mouse Shared Resource, Herbert Irving Comprehensive Cancer Center, Columbia University Irving Medical Center, New York, NY 10032 USA; 8 Division of Oncology, Department of Medicine, University of North Carolina at Chapel Hill, Chapel Hill, NC 27599 USA; 9 Department of Urology, University of North Carolina at Chapel Hill, Chapel Hill, NC 27599 USA; 10 Herbert Irving Comprehensive Cancer Center, Columbia University Irving Medical Center, New York, NY 10032 USA; 11 New York-Presbyterian Hospital, Columbia University Irving Medical Center, New York, NY 10032 USA

**Keywords:** Cancer models, Tumour heterogeneity, Bladder cancer

## Abstract

*Pparg*, a nuclear receptor, is downregulated in basal subtype bladder cancers that tend to be muscle invasive and amplified in luminal subtype bladder cancers that tend to be non-muscle invasive. Bladder cancers derive from the urothelium, one of the most quiescent epithelia in the body, which is composed of basal, intermediate, and superficial cells. We find that expression of an activated form of Pparg (*VP16;Pparg*) in basal progenitors induces formation of superficial cells in situ, that exit the cell cycle, and do not form tumors. Expression in basal progenitors that have been activated by mild injury however, results in luminal tumor formation. We find that these tumors are immune deserted, which may be linked to down-regulation of Nf-kb, a Pparg target. Interestingly, some luminal tumors begin to shift to basal subtype tumors with time, down-regulating Pparg and other luminal markers. Our findings have important implications for treatment and diagnosis of bladder cancer.

## Abstract (web summary)

PPARg is differentially expressed in bladder cancer subtypes. Here, the authors show in mice that when an activated form of PPARg is expressed in basal bladder cells tumours do not form, however in the presence of injury the basal cells differentiate into luminal cells.

## Introduction

Bladder cancers are the 9th most common form of cancer worldwide, and the 4th most common among men (Sung et al., ACS 2021). Estimates from the CDC indicate that in 2021, there are ~83,730 new cases of bladder cancer (~64,280 in men and ~19,450 in women) in the United States and 17,200 deaths from bladder cancer (~12,260 men and ~4940 women), with smoking accounting for almost half (47%) of all cases in the United States^1^. Bladder cancers tumors arise from the urothelium, a water-proof barrier lining the urinary outflow tract that extends from the renal pelvis to the bladder. The urothelium protects against infection, damage from toxins and prevents the exchange of fluids. It is one of the slowest cycling epithelia in the body^2^, but undergoes a rapid sequence of exfoliation and regeneration in response to acute injury or infection. However, persistent or repeated damage and inflammation can lead to permanent changes, including loss of endogenous urothelial populations and bladder pain disease^3,4^, which has limited treatment options.

The urothelium is pseudostratified, containing two populations of basal cells; K14-Basal cells (K14+ K5+) that are rare and reside exclusively in the basal layer, and K5-Basal cells (K5+ K14−), which reside in the basal and suprabasal layers. Lineage studies suggest that K14-Basal cells are progenitors that can repopulate the urothelium de novo^5–9^, and are also cells of origin that can produce tumors^10–12^. Intermediate cells (I-cells) and superficial cells (S-cells), which reside in upper urothelial layers, express luminal markers, including Krt20, Krt18, and Upks. I-cells (P63+ UPKs+) are direct progenitors that replace S-cells when they die off^7,13^. They are attached to the basement membrane by a long cytoplasmic tail, and they can either divide or undergo failed cytokinesis producing binucleated I-cells (2n+2n) that undergo endoreplication, doubling DNA content without entering mitosis to produce 4n+4n S-cells^9^. S-cells (UPK+ K20+ P63−) reside in the top layer; they are large, binucleated, and are critical for the synthesis and transport of uroplakins, a family of proteins that assemble into tough crystalline plaques that line the apical surface of the urothelium. Although S-cells are post-mitotic, they are functionally very active since apical plaque is continuously degraded and replaced in response to stretch, as the bladder fills and empties^14–18^.

Bladder cancers arise from the urothelium and were initially classified based on histology and clinical behavior^19^. In all, 51% of diagnosed cases are non-muscle-invasive bladder cancer (NMIBC) and 49% are muscle-invasive bladder cancer (MIBC)^20^. Approximately 10–20% of NMIBC cases eventually progress to MIBC^21^. More recent studies from a number of groups have identified distinct subtypes of bladder cancer based on mutations and transcriptional profiles that cluster with the luminal and Basal tumor types in MIBC. Approximately 47% of the cases are of the luminal subtype and 35% are of the Basal subtype^21–28^. These subtypes have grown in number and now include (LumP), luminal nonspecified (LumNS), luminal unstable (LumU), stroma-rich, basal/squamous (Ba/Sq), and neuroendocrine-like (NE-like)^21^. Tumors with a basal/squamous subtype express a set of markers including KRT14, KRT5, CD44, and KRT6A. Basal/squamous tumors are generally immune infiltrated and responsive to immune checkpoint inhibitors^21,23,29,30^. Bladder cancer of the luminal subtype expresses a set of markers found in urothelial I-cells and S-cells, including GATA3, FOXA1, and PPARG^23,31,32^. Luminal tumors are comparatively less invasive, but generally grow back after resection, and also tend to be immune poor andtend not to respond well to immune checkpoint blockers^21,33–35^.

PPARG-dependent transcription is important for a wide range of functions in different cell types, including adipogenesis, metabolism, and immunity (reviewed in refs. ^36,37^). PPARG agonists have profound effects on urothelial differentiation and *PPARG* mutations and amplifications contribute significantly to bladder cancers. *PPARG* is a member of the nuclear receptor superfamily of ligand-activated transcription factors that bind to response elements in regulatory regions of genes^37,38^. PPARG regulates transcription by forming heterodimers with RXR, a second nuclear receptor family member. PPARG/RXR heterodimers are activated when PPARG is bound by natural ligands (fatty acids and prostaglandins) or synthetic ligands including troglitazone and rosiglitazone. PPARG/RXR heterodimers bind to peroxisome proliferator response elements, and without ligand, are maintained in an inactive state, in complexes with co-repressors (NCOR2, SMRT). Ligand binding induces a conformational change in the PPARG/RXR heterodimer causing the release of co-repressors and the recruitment of co-activators [CREBBP, PPARGC1A, and HAT^39–41^].

Studies in knockout mice indicate that PPARG regulates urothelial differentiation both in the ureter and bladder^42,43^. In urothelial cell culture, PPARG agonists troglitazone and rosiglitazone in combination with an EGFR inhibitor, suppress squamous differentiation and induce expression of luminal markers, including *Upk1*a, *Upk2*, and *Krt20*^44,45^.

*PPARG* mutations and genomic alterations are common in bladder cancer. PPARG expression is downregulated in basal/squamous subtype tumors, suggesting that loss of signaling may promote bladder tumor formation. To address this, we previously generated mice lacking Pparg throughout the urothelium using the *ShhCre* driver. These studies revealed a number of profound changes in the urothelium, including squamous metaplasia and loss of endogenous urothelial populations, likely owing to alternations in the differentiation program of K14-Basal progenitors that produced squamous epithelial cells instead of urothelial cells. These observations suggest that Pparg is normally important for the specification of K14-basal cells, however, inactivation of *Pparg* alone during homeostasis is not sufficient to drive bladder cancer^43^.

Activation of Pparg-dependent transcription either owing to mutations in its binding partner RXR, or amplification of the *PPARG* gene occur in 20–25% of luminal tumors^46^. These observations prompted us to examine whether gain-of-function mutations in PPARG could induce luminal subtype bladder cancer in mice. To do this, we inserted a cassette containing the HSV VP16 activator fused to the amino-terminal of Pparg1^47^ into the *Rosa26* locus where it is activatable in cells expressing Cre recombinase.

The urothelium is an epithelial barrier that extends from the renal pelvis to the urethra. This stratified epithelium is nearly quiescent but can rapidly regenerate in response to injury. Here we show that *Pparg* signaling drives a luminal differentiation program in the urothelium during homeostasis as well as in tumor formation. Expression of constitutively active *VP16;Pparg* in basal progenitors during homeostasis drives them to differentiate into luminal (I-cells/S-cells) in situ; however, newly formed luminal cells are post mitotic, and do not form tumors. Expression of *VP16;Pparg* in basal progenitors that have been injured by a short exposure to BBN, a carcinogen found in tobacco smoke, results in activation of the basal population, which differentiate into luminal tumors, whereas in controls lacking *VP16;Pparg*, basal subtype tumors form. Injury of basal urothelial progenitors is accompanied but increased proliferation, a robust inflammatory response, and upregulation of *Krt6a* and *Krt16*, defensins that are not expressed in the healthy urothelium; a state that is quite similar to that observed in activated basal progenitors in the skin and airways^48–50^. Our findings suggest that basal cell activation may be a critical step in the genesis of urothelial carcinoma. Interestingly, we observed a shift at the base of some luminal lesions that increased with time, where tumor cells downregulate luminal markers and begin to express basal markers, suggesting that these tumors can undergo a luminal to basal shift. Together our studies provide a model for studying luminal tumor formation in vivo and may also shed light on the mechanisms underlying tumor evolution, a phenomenon that has been reported in recent studies^51,52^.

## Results

### *Pparg* activation in basal cells induces an S-cell differentiation program

**Fig. 1:**
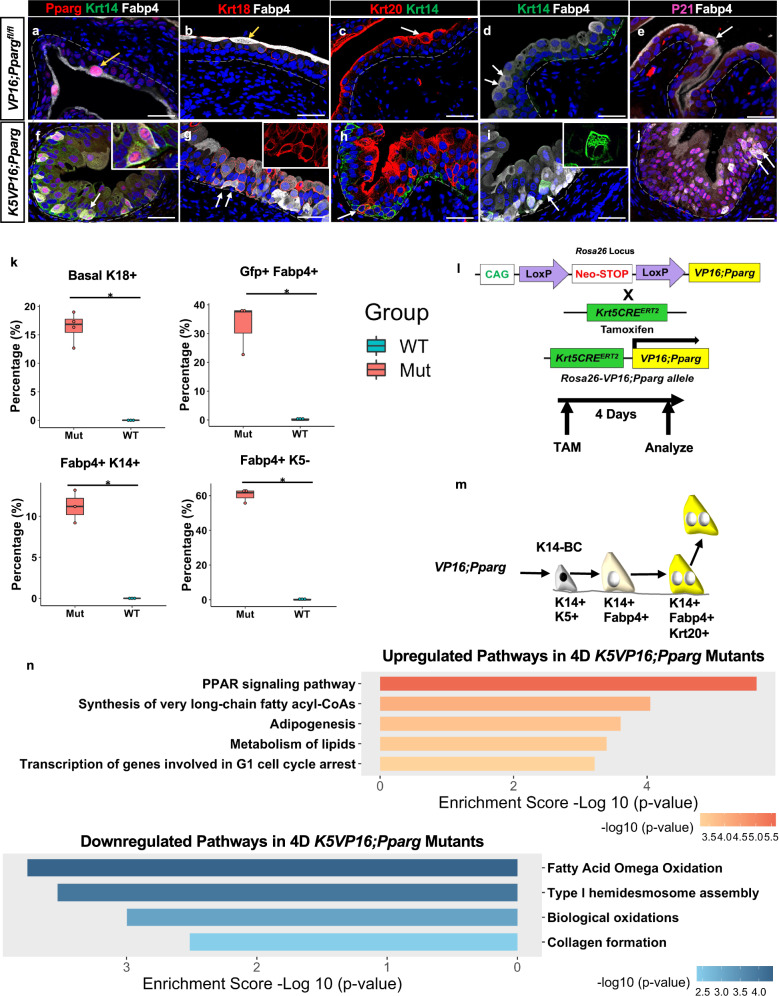
Expression of VP16;Pparg in basal cells induces an S-cell differentiation program. **a**–**j** Immunostaining showing expression of Pparg, Krt14, and Fabp4 in the urothelium of *VP16;Pparg* control (**a**) and *K5VP16;Pparg* mutants (**f**). Expression of Krt18 and Fabp4 in the urothelium of control (**b**) and a *K5VP16;Pparg* mutant (**g**). Expression of Krt20 and Krt14 in the urothelium of control (**c**) and a *K5VP16;Pparg* mutant (**h**). Expression of Krt14 and Fabp4 in the urothelium of control (**d**) and *K5VP16;Pparg* mutant (**i**). Expression of P21 and Fabp4 in the urothelium of control (**e**) and a *K5VP16;Pparg* mutant (**j**). **k** Quantification of the percentages of Basal cells undergoing luminal differentiation in *VP16;Pparg* controls (*n* = 3) versus *K5VP16;Pparg* mutants 4 days (*n* ≥ 3) after tamoxifen induction: expression of cell-type-specific markers in mutants and controls: Krt18 in the basal layer (*p* = 0.021), Gfp and Fabp4 in the basal layer (*p* = 0.038), Fabp4 and K14 (*p* = 0.031), Fabp4 but not K5 (*p* = 0.038). Box plots display minima, maxima, and interquartile range (IQR). Significance was calculated by one-sided Mann–Whitney *U* test. *p ≤ 0.05. **l** Schematic showing the mouse models and timing of analysis. The *VP16;Pparg* cassette was inserted in the *Rosa26 Locus* to generate *VP16;Pparg* mutant mice, where expression is activatable in cells expressing Cre recombinase. *VP16;Pparg* mice were then crossed with the *Krt5Cre*^*ERT2*^ line, which drives Cre-dependent recombination in Basal cells after Tamoxifen induction, activating the expression of the transgene. Tamoxifen was administered 3× over the course of 1 week, and bladders were harvested 4 days after the last Tamoxifen induction. **m** Schematic of the S-cell differentiation program induced by *VP16;Pparg* expression in basal cells. **n** Upregulated and downregulated pathways from RNA-seq analysis of controls and *K5VP16;Pparg* mutants 4 days after Tamoxifen induction. *p* values were calculated by hypergeometric test and corrected for multiple testing. Yellow arrows in **a**–**e** denote superficial cells. White arrows in **f**–**j** denote mutant cells undergoing a basal to luminal shift. Scale bars, 50 μm. Source data are provided as a Source Data file.

The *PPARG* gene is amplified in tumors of the luminal subtype, which also express high levels of FABP4, a direct transcriptional target of *PPARG*^23^. This suggests that *PPARG* is both overexpressed and transcriptionally active, most likely by endogenous ligands. To generate a gain-of-function model that mirrors the increased *PPARG* activity in luminal tumors, we generated mice harboring a constitutively active form of Pparg1 that is tamoxifen-inducible. We inserted a cassette containing the HSV *VP16* activator fused to the N-terminal of Pparg1 into the *Rosa26* locus where it is activatable in cells expressing Cre recombinase [Fig. 1l^53^]. Unlike endogenous *Pparg*, *VP16;Pparg* is transcriptionally active without ligand binding^47,53^. K14-Basal cells have been shown to be progenitors that can produce tumors in mice^10^. To target this population, *VP16;Pparg* mice were crossed with the *Krt5Cre*^*ERT2*^ line generating *Krt5Cre*^*ERT2*^*;VP16;Pparg* mutants (hereafter, referred to as *K5VP16;Pparg* mutants), in which tamoxifen-inducible Cre driven by the K5 promoter drives recombination in the basal cell population^54^.

Tamoxifen was administered intraperitoneally three times over the course of 1 week which induced recombination in 80% of basal cells. To determine whether the VP16;Pparg mutant protein was transcriptionally active, we analyzed the distribution of Fabp4, a direct transcriptional target of *Pparg* in *K5VP16;Pparg* mutants and *VP16;Pparg*^*fl/fl*^ controls 4 days after Tamoxifen induction. In controls Fabp4 and Pparg were co-expressed in S-cells as expected (Fig. 1a), however in *K5VP16;Pparg* mutants, Fabp4 and Pparg were expressed both in S-cells in the luminal layer and in K14-basal cells in the basal layer (Fig. 1f, k, Supplementary Fig. 1r) indicating that the *VP16;Pparg* transgene is active. Further analysis revealed Krt18 and Krt20, which are selectively expressed in S-cells of controls (Fig. 1b,c), co-localized with Fabp4 and Pparg in K14-BC-positive cells in the basal layer (Fig. 1g–k), suggesting that the *VP16;Pparg* transgene is inducing K14-Basal cells to differentiate into S-cells in situ. Consistent with this, we observed binucleated cells co-expressing K14 and Fabp4 in the basal layer suggesting that this newly formed population has undergone failed cytokinesis, a process observed when I-cells differentiate into S-cells, which normally occurs in the luminal layers of the urothelium (Fig. 1d,i,^9^).

Lineage tracing confirmed that K14+/Fabp4+/Krt20+ cells that formed in *K5VP16;Pparg* mutants were derived from basal cells. The *R26RmTmG* reporter^55^ was introduced into mutants and controls to generate *K5VP16;Pparg;mTmG* mice and *VP16;Pparg*^*fl/fl*^;*mTmG* mice, respectively. In controls, Gfp-expression was confined to the basal layer as expected (Supplementary Fig. 1m–p), whereas in *K5VP16;Pparg* mutants, Gfp-expressing cells which were observed in the basal layer 1 day after tamoxifen induction, expanded to encompass the entire urothelium by one 1 month (Supplementary Fig. 1q–t)

To identify changes in the transcriptional program of basal cells induced by expression of the *VP16;Pparg* transgene, we performed bulk RNA-seq analysis on Gfp+ cells collected from *K5VP16;Pparg;mTmG* mutants and *K5*;*mTmG* controls (Supplementary Fig. 5). Pathway analysis revealed upregulation of *Pparg* signaling in basal cells of mutants, confirming that the transgene was active (Fig. 1n). This analysis also revealed upregulation of S-cell markers in basal cells of mutants compared with controls, whereas *Krt5*, *Col17a1*, and *Trp63* which are highly expressed in basal cells of controls were downregulated (Supplementary Fig. 1y), which was confirmed by immunostaining (Fig. 1k, Supplementary Fig. 1u–x). Col17a1 is required for anchoring basal cells to the cell membrane, hence its downregulation may enable newly S-Cells to move from the basal layer up to the superficial layer of the urothelium where S-cells normally reside. RNA-seq analysis also revealed changes in cell cycle genes including p21 (*Cdkn1a*), which was upregulated in basal cells from *K5VP16;Pparg* cells compared to *VP16;Pparg* controls and confirmed by immunostaining (Fig. 1e, j), indicating that expression of the *VP16;Pparg* transgene-induced S-cell like daughters to leave the cell cycle, as is the case with endogenous S-cells. Together these results suggest that the expression of the *VP16;Pparg* transgene in basal cells alters the normal differentiation program, resulting in the production of S-like cells in situ.

Squamous metaplasia in the airways, bladder, and prostate is induced by Vitamin A-deficiency^56,57^. In this case, the urothelium is populated by cells expressing markers found in the skin, Krt13, Krt1, Krt10, similar to the phenotype induced in the urothelium in *Pparg* mutants, which is characterized by an increase in K14-progenitors accompanied by a decrease in I-cells and S-cells^43^. Retinoids have also been shown to be required for urothelial differentiation and regeneration, specifically for the formation of I-cells and S-cells^13^. We observed increased retinoid signaling in *K5VP16;Pparg* mutants compared with *VP16;Pparg*^*fl/fl*^ controls (Supplementary Fig. 1z). Upregulated genes include *Rbp4*, a lipocalin family member that transports retinol (the inactive form of Vitamin A) from the liver to tissues, *Stra6*, a receptor that transports retinol into the cell, *Rdh11* which synthesizes retinaldehyde from retinol in the first step of RA synthesis and *Aldh1a3*, a retinaldehyde dehydrogenase that converts retinaldehyde to RA, in the second step of RA synthesis [Supplementary Fig. 1z^58,59^]. These data suggest that *Pparg*-dependent RA-signaling is likely important for differentiation of luminal cell types, and for suppression of squamous differentiation.

Consistent with the known role of *Pparg* as a regulator of fatty-acid transport and metabolism, we observed an increase in genes important in lipid metabolism (Fig. 1n). These include *Scd1, Elovl, Acsl4, Cpt1, Fabp5, Fabp1*, *Lpl*, and *Cd36*. Genes involved in pancreatic secretion, protein digestion and absorption, digestion of dietary lipid, and triacylglycerol degradation were downregulated (Fig. 1n). These pathways are similarly altered in non-alcoholic fatty liver disease (NAFLD)^60–64^, which is thought to be linked to high-fat diet-induced *Pparg* signaling^65^. Oil-Red-O staining of mutants 1 day, 4 days, and 1 month after Tamoxifen induction, revealed neutral triglyceride and lipid accumulation in mutant S-cells at 1 month, but staining was not detectable at earlier stages or in controls (Supplementary Fig. 1a–l). These observations raise the possibility that the urothelium may respond to a high-fat diet in a similar manner as observed in hepatocytes in steatosis, which turn on an adipocyte differentiation program in response to increased *Pparg* signaling.

Together, these studies suggest that constitutive activity of *Pparg* in K14-basal cell progenitors induces the formation of S-cell daughters instead of K5-Basal cells and I-cells, as normally occurs, a process that may depend on RA-signaling. With time, these S-cells appear to turn on an adipocyte differentiation program as occurs in NAFLD. These VP16;Pparg-expressing S-cells are post-mitotic however and hence did not form tumors.

### Short-term treatment with carcinogens primes K14-basal cells for tumor formation by inducing an activated state

**Fig. 2:**
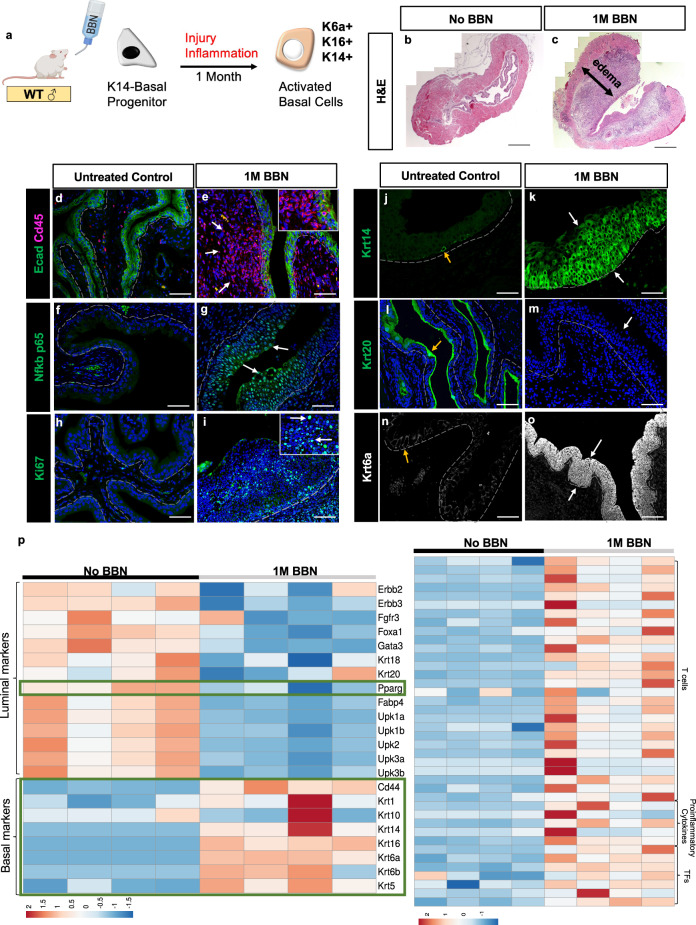
Short BBN treatment induces a wound-healing response in the urothelium that primes basal cell progenitors for tumorigenesis. **a** Schematic representation showing activation of K14-progenitors. **b**–**c** Histology of bladders from untreated mice (**b**) and from mice treated with BBN for 1 month (**c**); the black double-headed arrow denotes edema. Scale bars, 200 μm. **d**–**i** Immune infiltration visualized by Cd45 staining in bladders from untreated controls (**d**) and mice treated with BBN for 1 month (**e**). Expression of p65 in controls (**f**) and in mice treated with BBN for 1 month (**g**). Expression of Ki67 in controls (**h**) and in bladders from mice treated with BBN for 1 month (**i**). White arrows denote cells positive for Cd45 (**e**), p65 (**g**), and Ki67 (**i**) in bladders from BBN-treated animals. The inserts in **e** and **i** show images at higher magnification. **j**–**o** Loss of normal urothelial populations and gain of squamous populations in bladders from a mouse treated with BBN for 1 month. Krt14 expression in control (**j**) and in urothelium from a mouse treated with BBN for 1 month (**k**). Yellow arrows in **j** denote a Krt14-expressing cell. Krt20 expression in a control (**l**) and in the urothelium from a mouse treated with BBN for 1 month (**m**). The yellow arrow in **l** denotes a Krt20-expressing S-cell. The white arrow in **m** denotes the loss of Krt20-positive S-cells after BBN treatment. Krt6a expression in control (**n**) and in urothelium from a mouse treated with BBN for 1 month (**o**). The yellow arrow in **n** denotes the absence of detectable Krt6a expression in the untreated urothelium. White arrows in **o** denote activated basal cells expressing Krt6a, a squamous marker not present in the healthy urothelium. Scale bars, 50 μm. **p** Heatmaps of luminal/basal gene signatures and immune cell signatures generated from pathway analysis of RNA-seq performed on the urothelium of control and BBN-treated bladders.

Currently, the most established system for modeling MIBC in mice is *N*-butyl-*N*-(4-hydroxybutyl)-nitrosamine (BBN)^66^. BBN is a nitrosamine that is metabolized in the liver to *N*-butyl-*N*-(3-carboxypropyl)nitrosamine, which is found in tobacco products^66–68^. Five months of exposure to BBN induces basal subtype tumors, however short exposure induces a potent inflammatory response that resolves by ~1 month and recedes as tumors form^69^. An interesting possibility is that this transient response to BBN might be important for priming urothelial cells, which are largely quiescent, to re-enter the cell cycle and produce tumors. To address this question, BBN was administered to wild-type mice in water for 4 weeks, then we analyzed the urothelium from treated and untreated mice to determine the effects on urothelial populations as well as immune infiltration (Fig. 2a). Histological analysis revealed edema and immune infiltration in the bladder of BBN-treated mice which was not observed in controls, indicating the presence of inflammation (Fig. 2b, c). Consistent with this, immunostaining revealed significant leukocyte infiltration in BBN-treated mice, as well as upregulation of p65 subunit of Nf-kb, a complex, that controls both innate and adaptive immunity (Fig. 2d–g). These observations were confirmed by RNA-seq analysis, which revealed upregulation of T-cell markers (Cd4 and Cd8), proinflammatory cytokines (*Il1a*, *Il6*, *Il18*, *IFNg*, and *TNFa)*, and transcription factors, including *Nfkb, Foxp3*, *Stat3* and *Jun*, *Batf*, and *Fosl1*, which are *Ap1* family members that are important regulators of the immune response^70^ (Fig. 2p, Supplementary Fig. 2a).

Further analysis by immunostaining and RNA-seq revealed widespread proliferation in the urothelium of BBN-treated compared with untreated controls, where proliferating cells were rare (Fig. 2h, i). This short BBN treatment also caused profound alterations in urothelial populations: K14-Basal cells, which are rare in controls and are confined to the basal layer, now populated most of the urothelium in BBN-treated mice (Fig. 2j, k). Consistent with the massive expansion of the K14-population, we observed depletion of I-cells and S-cells in BBN-treated mice, evidenced by downregulation of Krt20 (Fig. l, m) as well as *Fgfr3*, *Foxa1*, *Gata3*, *Krt18,* and *Upks* (Fig. 2p). On the other hand, BBN exposure induced upregulation of *Krt6a* which is barely detectable in the urothelium of healthy mice (Fig. 2n, o) as well as keratins expressed in squamous epithelia including *Cd44*, *Krt1*, *Krt16*, *Krt6b,* and *Krt5* (Fig. 2p). In addition to BBN, we found that exposure to another urotoxin cyclophosphamide^71^ induces similar activation of the K14-Basal cells (Supplementary Fig. 2b–m). K14-progenitors in the skin and airways, which are also barriers, upregulate *Krt14*, *Krt16,* and *Krt6a* and undergo squamous differentiation. Hence, this activation state may be a conserved reaction to injury, perhaps maintaining the barrier by regenerating a layer of squamous tissue in response to injury.

### *VP16;PPARG* expression in activated basal cells induces luminal tumor formation

**Fig. 3:**
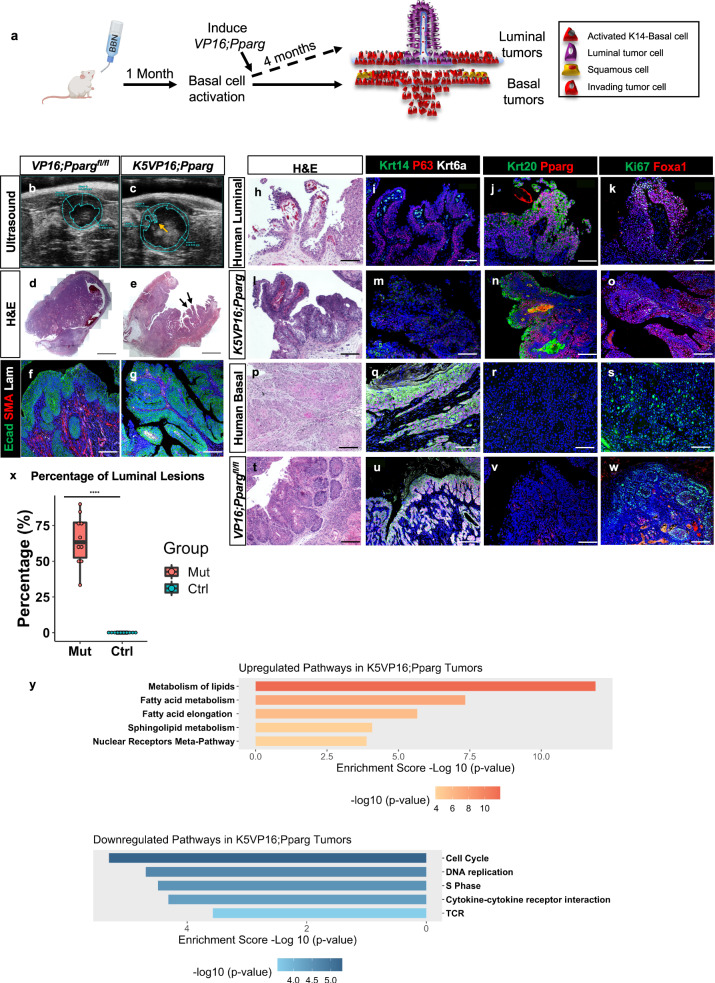
Activation of Pparg in K5VP16;Pparg mice produces luminal bladder tumors. **a** Schematic showing time-course of tamoxifen and BBN treatment used in experiments with *K5VP16;Pparg* mice and controls. **b**–**g** Ultrasound images of *VP16;Pparg*^*fl/fl*^ control bladders (**b**) and a *K5VP16;Pparg* mutant (**c**) 4 months after Tamoxifen induction. Yellow arrow in **c** denotes a lesion protruding into the lumen. H&E images of bladders from a *VP16;Pparg*^*fl/fl*^ control (**d**) and a *K5VP16;Pparg* mutant (**e**) 4 months after Tamoxifen induction. Double black arrows in (**e**) denote high grade papillary lesions. Expression of E-cadherin, smooth muscle actin, and laminin in a *VP16;Pparg*^*fl/fl*^ control (**f**) and in a *K5VP16;Pparg* mutant (**g**). **h**–**w** An H&E image of human luminal tumor (**h**), a *K5VP16;Pparg* mutant tumor (**l**), a human basal tumor (**p**), and a *VP16;Pparg* control basal tumor (**t**). Expression of Krt14, P63, and Krt6a in a human luminal tumor (**i**), a *K5VP16;Pparg* mutant tumor (**m**), a human basal tumor (**q**), and a *VP16;Pparg* control basal tumor (**u**). Krt20 and Pparg expression in a luminal tumor (**j**), a *K5VP16;Pparg* mutant tumor (**n**), a human basal tumor (**r**), and a *VP16;Pparg* control basal tumor (**v**). Ki67 and Foxa1 expression in a luminal tumor (**k**), a *K5VP16;Pparg* mutant tumor (**o**), a human basal tumor (**s**), and a *VP16;Pparg* control basal tumor (**w**). **x** Quantification of the percentage of luminal tumors observed in *VP16;Pparg* controls (*n* = 10) and *K5VP16;Pparg* mutants (*n* = 10). Box plots display minima, maxima, and interquartile range (IQR). Significance was calculated by a one-sided Mann–Whitney *U* test. ****p = 3.14e-05. **y** Upregulated and downregulated signaling pathways based on RNA-seq analyses of *K5VP16;Pparg* mutant tumors compared to *VP16;Pparg*^*fl/fl*^ basal control tumors 4 months after tamoxifen induction. *p* values were calculated by hypergeometric test and corrected for multiple testing. Scale bars in **d**–**e** 200 μm. Scale bars in **f**–**w**, 100 μm. Source data are provided as a Source Data file.

Long-term BBN treatment of wild-type mice results in basal subtype tumors^35,72^; however, a short BBN exposure activates the urothelium, which becomes proliferative and is populated almost exclusively with K14-Basal cells compared with untreated mice (Fig. 2h–o). We tested whether expression of the *VP16;Pparg* protein after activation could alter the pathway of tumor formation from basal subtype to luminal subtype (Fig. 3a). *K5VP16;Pparg* mutants and controls were exposed to BBN for 1 month, after which tamoxifen was administered intravesically under ultrasound guidance. Ultrasound analysis after 4 months revealed thickening of the bladder wall in controls and protrusions in the lumen of mutants (Fig. 3b, c). Hematoxylin and eosin stain (H&E) revealed papillary-like structures in *K5VP16;Pparg* mutants and invasive lesions in *VP16;Pparg*^*fl/fl*^ controls (Fig. 3d, e). The pathological evaluation suggested that lesions in *K5VP16;Pparg* mutant mice were consistent with a human papillary luminal subtype of bladder cancer, whereas tumors in controls lacking the transgene presented features of the human basal/squamous subtype, as expected. Lesions in the *K5VP16;Pparg* mutant bladders were enriched with Ta and T1 (exophytic) lesions (Fig. 3l), whereas control bladders without the *VP16;Pparg* transgene contained invasive T3 (basal) lesions (Fig. 3t, x). Laminin and smooth muscle actin (SMA) staining allowed us to clearly visualize the fibrovascular core of luminal lesions, a hallmark of papillary (luminal) tumors while in controls, vasculature was widespread (Fig. 3f, g). To confirm that lesions derived from basal cells in mutants and controls, we analyzed bladders from *K5VP16;Pparg;mTmG* mutants and *K5;mTmG* controls 4 months after tamoxifen induction when lesions were clearly visible. In both cases, lesions were almost completely Gfp-positive (Supplementary Fig. 3a, b), indicating that they are derived from Gfp+ basal cells.

Comparison of BBN-induced lesions in *K5VP16:Pparg* luminal lesions with human luminal tumors from patients revealed a similar branched exophytic structure and a fibrovascular core in both. Conversely, BBN-treated control lesions were invading into the submucosa and muscle, similar to the human basal tumor (Fig. 3h, l, p, t). Immunostaining of human and mouse lesions with markers expressed in basal subtype lesions (Krt14 and Krt6a) revealed robust expression in the BBN-treated controls and human basal tumor, whereas there was little if any expression in lesions from mouse *K5VP16;Pparg* mutants or luminal lesions from human patients (Fig. 3i, m, q, u). Analysis with markers expressed in luminal subtype tumors (Krt20, Pparg, Foxa1) revealed little expression in control BBN-induced basal lesions and human basal tumors, whereas expression was prominent in luminal human tumors and *K5VP16;Pparg* tumors (Fig. 3j, k, n, o, r, s, v, w). Expression of Ki67, which marks proliferating cells, was distinct in basal and luminal tumors. In basal tumors, Ki67 expression was in cells adjacent to the basement membrane of lesions, while in luminal tumors proliferating cells were scattered (Fig. 3k, o, s, w).

RNA-seq analysis of laser-captured cells from *K5VP16;Pparg* and control mice revealed co-clustering with genes expressed in tumors with luminal and basal subtypes, respectively. Comparison of the transcriptomes of BBN-induced tumors with subtypes from Cancer Genome Atlas (TCGA) human tumors (*n* = 408) and known luminal bladder cancer model *Upk3a-CreERT2; Trp53L/L; PtenL/L; Rosa26LSL-Luc* (UPPL) tumors and controls reveal that the *K5VP16;Pparg* mutant lesions co-clustered with luminal and luminal Papillary TCGA samples, which express a set of markers including *Pparg, Foxa1, Krt18, and Krt20* (Supplementary Fig. 3e). Control basal lesions from BBN-treated *VP16;Pparg*^*fl/fl*^ mice co-clustered with the Basal Squamous TCGA samples, which express a set of markers including *CD44, Krt5, Krt14, and Krt6a* (Supplementary Fig. 3e). Pathway analysis revealed upregulation of genes related to metabolism in *K5VP16;Pparg* mutant lesions (Fig. 3y), which is not surprising given the known role of *Pparg* as a regulator of mitochondrial biogenesis and fatty-acid metabolism. Oil-red-O staining showed accumulation of lipid droplets in the urothelium of *K5VP16;*Pparg1 month after Tamoxifen induction, however, we did not observe lipid droplets in the *K5VP16;Pparg* mutant luminal tumors (Supplementary Fig. 3c, d). Pathways related to cell cycle, T-cell activation, and cytokine signaling are downregulated in the *K5VP16;Pparg* mutant lesions, suggesting the immune response is dampened in the *Pparg* activated luminal tumors (Supplementary Fig. 3e). Taken together, these findings suggest activating *Pparg* in activated basal cells can induce basal tumors to adopt a luminal fate.

### *Pparg* induced luminal tumors are immune cold

**Fig. 4:**
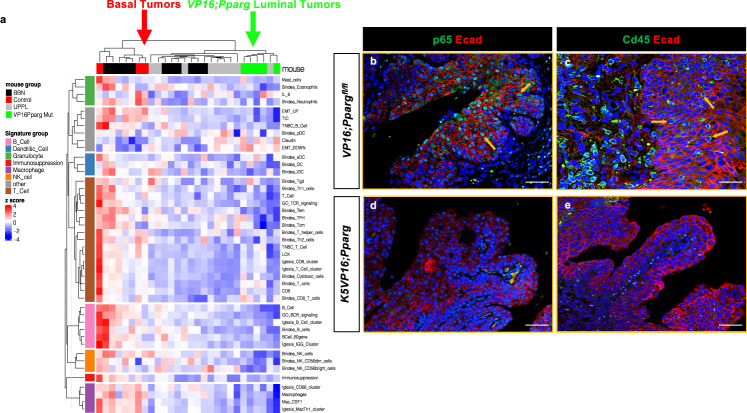
K5VP16;Pparg tumors are immune cold. **a** Immune gene signatures of *K5;mTmG* control tumors, *K5;VP16;Pparg;mTmG* mutant tumors, tumors induced by BBN, and UPPL primary tumor samples. Expression of p65 and E-cadherin in controls (**b**) and in *K5VP16;Pparg* mutant tumors (**d**). Expression of Cd45 and Ecad in controls (**c**) and in *K5VP16;Pparg* mutant tumors (**e**). Yellow arrows in **b** and **c** denote p65 and Cd45-expressing cells in control tumors. Scale bars, 100 μm.

An important distinction in the classification of tumors is whether the tumor is immune “hot” or immune “cold.” Hot tumors are characterized by increased immune cell trafficking, an abundance of inflammatory cytokines and antigen-presenting cells, increased T-cell activation, and increased major histocompatibility complex expression. Cold tumors have low to no immune cell trafficking, impaired T-cell activation, an abundance of myeloid-derived suppressor cells, and regulator T cells that release immunosuppressive cytokines^73,74^. Human basal and luminal MIBCs demonstrate varying degrees of immune infiltration and respond differently to checkpoint inhibitors. Immune infiltration is mostly associated with the basal/squamous and stroma-rich subtypes of MIBC, whereas the luminal subtypes are often immune cold^21^, suggesting that the tumor microenvironment in bladder cancer is driven by subtype-specific differences. However, whether the immune exclusion phenotype is due to active immune suppression in the luminal subtypes largely unknown. *Pparg* is known to regulate a number of immune responses, including NF-kB suppression and interactions with the AP1 pathways^75–78^. Unsupervised clustering of *K5VP16;Pparg* mutants and *VP16;Pparg*^*fl/fl*^ control tumors using previously established immune gene signature demonstrated overall low levels of the immune response, similar to the UPPL luminal bladder cancer model (Fig. 4a,^35^). In contrast, the control tumors showed very active immune signatures (Fig. 4a). To further investigate these findings, we compared expressions of Nf-kb subunit p65 and Cd45 in *K5VP16;Pparg* mice and *VP16;Pparg*^*fl/fl*^ controls (Fig. 4b–e). Immunostaining of control basal tumors revealed widespread nuclear expression of p65, indicating that Nf-kb is activated throughout the tumor. In addition, we observed numerous infiltrating Cd45+ leukocytes (Fig. 4c, e). Analysis of p65 expression in *K5VP16;Pparg* mutant tumors, reveal little if any nuclear expression, suggesting that Nf-kb is inactive in the mutants. Likewise, we did not observe infiltrating immune cells in these lesions based on Cd45 expression. *Pparg* actively represses Nf-kb expression, suggesting that Pparg is likely to be important for inducing the immune cold phenotype in luminal tumors.

### A subset of luminal tumors in *K5VP16;Pparg* mutants undergo a shift toward the basal subtype

**Fig. 5:**
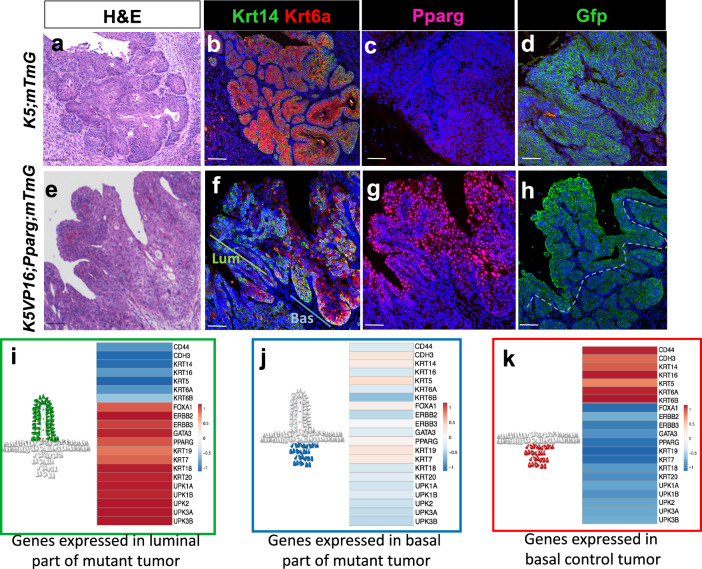
K5VP16;Pparg;mTmG luminal tumors develop basal domains. **a**–**k** H&E images of a *K5;mTmG* control tumor (**a**) and a *K5VP16;Pparg;mTmG* mutant tumor (**e**) 4 months after induction. Expression of Krt14 and Krt6a in a *K5;mTmG* control tumor (**b**) and *K5VP16;Pparg;mTmG* mutant tumor (**f**) 4 months after induction. Expression of Pparg in a *K5;mTmG* control tumor (**c**) and a *K5VP16;Pparg;mTmG* mutant tumor (**g**) 4 months after induction. Expression of Gfp in a *VP16;Pparg;mTmG* control tumor (**d**) and in a *K5VP16;Pparg* mutant tumor (**h**) 4 months after Tamoxifen induction. **i**–**k** Heatmaps and schematic representation showing tumor evolution. The luminal portion of a *K5VP16;Pparg* mutant tumor is shown in green (**i**) and the basal domain is depicted in blue (**j**). **k** shows a schematic representation of a control basal tumor, which is depicted in red. Scale bars, 100 μm.

During the course of our analysis, we observed a domain in a few luminal tumors that appear to be more basal in character than luminal (Fig. 5a, e). Mixed subtypes were observed n 15/70 lesions in *K5VP16;Pparg* mutants 4 months after Tamoxifen induction (Table 1, Supplementary Fig. 4a). However, these mixed subtype tumors were rare in animals analyzed 1 month earlier, where only 1/28 lesions displayed a mixed subtype (Table 1, Supplementary Fig. 4a), suggesting that the shift increases with time. Analysis with Krt6a and Krt14, markers that stain basal subtype tumors, revealed robust expression throughout basal lesions in controls that were exposed to BBN for 5 months but do not express the *VP16;Pparg* transgene (Fig. 5b). Analysis of Krt6 and K14 expression in tumors with mixed histology revealed little staining in the luminal compartment (Fig. 5f, green line), whereas staining was increased in the basal-like compartment (Fig. 5f, blue line). *Pparg* expression was undetectable in basal control tumors (Fig. 5c), whereas expression was maintained at high levels in the mutant luminal upper domain (Fig. 5g). In the basal-like portion of these mixed lesions, however, levels were lower compared with the luminal domain (Fig. 5g).

**Table 1:** Quantification of tumor class in *VP16;Pparg* controls and *K5VP16;Pparg* mutants.

Time point	Sample	No. of luminal lesions		No. of basal lesions	Time point	Sample	No. of luminal lesions		No. of basal lesions
		Luminal only	Luminal with basal bottom				Luminal only	Luminal with basal bottom	
*4 Months K5VP16;Pparg*	Het 72	5	1	2	*4 months control*	WT 5	0	0	2
	Het 74	8	1	1		WT 3	0	0	5
	Het 51	6	3	6		WT 88	0	0	1
	Het 1	18	4	4		WT 54	0	0	3
	Het 53	2	4	3		WT 55	0	0	1
	Het 56	1	0	1		WT 2	0	0	3
	Het 57	3	0	3		WT 83	0	0	5
	Het70	5	1	4		WT 20	0	0	2
	Het 9	1	0	2		WT 19	0	0	1
	Het 75	6	1	2		WT 56	0	0	2
	Total	55	15	28		Total	0	0	25
*3 Months K5VP16;Pparg*	Het 44	8	1	3	*3 months control*	WT61	0	0	3
	Het 62	6	0	3		WT58	0	0	4
	Het 50	2	0	0		WT59	0	0	4
	Het 4	7	0	1		WT 5	0	0	4
	Het 7	4	0	3		WT81	0	0	3
						WT22	0	0	2
	Total	27	1	10		Total	0	0	20
*2 Months K5VP16;Pparg*	Het70	4	0	0	*2 months control*	WT50	0	0	1
	Het 56	1	0	0		WT49	0	0	1
	Het 62	2	0	0		WT40	0	0	2
	Total	7	0	0		Total	0	0	4
*1 Month K5VP16;Pparg*	Het 8	0	0	0	*1 month control*				
	WT 66	0	0	0					
	Het 50	0	0	0		WT 70	0	0	0
	Het 10	0	0	0		WT 79	0	0	0
	Total	0	0	0		Total	0	0	28

Overall classification and quantification of lesions observed in *VP16;Pparg* controls and *K5VP16;Pparg* mutants at the indicated timepoints. Pathologic classification is based on the analysis of H&E-stained samples.

Lineage tracing of *K5;mTmG* controls, revealed Gfp expression throughout Basal subtype tumors (Fig. 5d). Analysis of *K5VP16;Pparg*;*mTmG* mice revealed Gfp-labeling both in the luminal domain and in the basal-like domain of mixed subtype tumors, indicating that both luminal and basal-like compartments arose from cells expressing the *K5VP16;Pparg*;*mTmG* transgene (Fig. 5h). The observations that (i) number of tumors with the mixed basal/luminal subtype increases with time, (ii) that these tumors express *Ppar*g both in luminal and basal-like domains (iii) that both luminal and basal domains are Gfp positive, suggests that lower basal-like domain derived from the upper luminal tumor, and had begun to shift from a luminal to a basal subtype.

To further characterize changes in the mixed subtype lesions, we performed RNA-seq analysis using laser capture to collect tissue from the luminal and basal-like domains separately, as well as from basal subtype controls. Analysis of basal subtype controls revealed high levels of expression of basal subtype markers, including *CD44*, *Krt6a*, *Krt14,* and *Krt16*, whereas expression of luminal markers was low (Fig. 5k, Supplementary Fig. 4b). Analysis of the upper luminal domain of mixed subtype lesions revealed a pattern of expression typical of luminal tumors; basal markers were downregulated and luminal markers including *Pparg*, *Upks*, *Krt20*, *Gata3,* and *Foxa1* were upregulated (Fig. 5i, Supplementary Fig. 4b). Expression of most luminal markers was low in the basal-like portion of the tumor compared with the luminal upper portion (Fig. 5j, Supplementary Fig. 4b) while a subset of basal markers was upregulated, consistent with the results of immunostaining (Fig. 5j, Supplementary Fig. 4b). Pathway analysis revealed increases in genes important for metabolism and lipid synthesis, as well as glutathione-mediated detoxification, a likely response to BBN exposure and the Ppar signaling pathway. Genes upregulated in the basal portion of the mixed subtype tumors include those important for cell cycle regulation, T-cell signaling, and inflammation, which is consistent with the immune infiltrated phenotype of basal subtype tumors (Supplementary Fig. 4c). Taken together, our observations suggest that advanced luminal tumors lose Pparg expression, display increased expression of basal markers, and develop invasive basal domains. This shift in phenotype has recently been reported in patient-derived organoids^52^ as well as in human tumors^51^. We have established a mouse model that undergoes a phenotypic shift from a luminal subtype towards a basal subtype. Luminal tumors are often classified as NMIBC, however, muscle-invasive lesions develop in 10–20% of patients diagnosed with luminal NMIBC. An important question is whether the invasive lesions develop from the original luminal tumor in a similar way as observed here since luminal tumors are generally removed by transurethral resection of tumors (TURBT), which may leave behind the basal-like invasive portion of the tumor.

## Discussion

*Pparg* has the capacity to regulate numerous cellular functions, including metabolism, fatty-acid transport, and cell-type specification. *PPARG* expression is downregulated in the basal subtype of bladder cancer, and amplification of the *PPARG* gene or activating mutations in RXR, the PPARG binding partner, are present in ~17% of luminal tumors^22,79,80^, which has led to the suggestion that *PPARG* drives luminal tumor formation. The healthy urothelium is populated by a superficial layer of S-cells which are post-mitotic, layers of I-cells that can replace S-cells when they die off during homeostasis or acute infection, K5-Basal cells that reside in the basal and suprabasal layers, and K14-Basal cells, which are rare and are confined to the basal layer. Based on lineage studies with the Krt5CreERT2 driver, K14-basal cells have the capacity to regenerate the urothelium de novo^5,11,12^, and lineage studies together with a BBN model of carcinogenesis indicate that K14-Basal cells also give rise to bladder cancers^10^. Our studies indicate that Pparg has an important role in specifying the differentiation program of K14-Basal cells. Our previous studies suggest that loss of Pparg results in the impaired differentiation of I-Cells and S-cells, increased proliferation, and squamous metaplasia. In this case, the K14-basal population expands and produces squamous cell types instead of endogenous urothelial populations^43^. The studies described here indicate that active Pparg signaling can induce K14-Basal cells to differentiate into S-cells in situ and can also shift the differentiation program in basal subtype bladder cancer toward a luminal subtype. We also observe changes in the differentiation state of luminal tumors over time that suggest a luminal to basal shift is occurring. This shift is accompanied by downregulation of *Pparg* as well as other luminal markers. Whether loss of *Pparg* is the critical factor that induces these changes is an important question.

Cigarette smoking is a major risk factor for bladder cancer^1^. Several studies indicate that carcinogens in tobacco smoke induce the formation of DNA adducts that lead to DNA damage and point mutations^81,82^. Point mutations can lead to the production of neoantigens that are recognized as foreign by resident dendritic cells or macrophages triggering an inflammatory response. Expression of the *VP16;Pparg* transgene in K14-Basal cells during homeostasis induces a terminal differentiation program, however, we find that a short period of exposure to BBN, a carcinogen or cyclophosphamide that contains acrolein alters the urothelial microenvironment, inducing an inflammatory response that may prime K14-basal cells for tumor formation.

Studies in the skin and airways indicate that K14-basal cells, which are progenitors in both tissues, respond to injury or inflammation by taking on an activated state^48,49,83^. These cells produce squamous epithelial cells instead of epidermal cell types, which upregulate Krt6a and Krt16. We find a similar situation exists in the urothelium in response to a short period of carcinogen exposure. K14-basal progenitors normally self-renew and produce K5-Basal cells that populate the basal and suprabasal layers, and I-cells daughters that differentiate into S-cells. We find that 1 month of BBN exposure or repeated exposure to cyclophosphamide induces an activation state in K14-basal cells similar to that observed in the epidermis and airways. The K14-basal population becomes proliferative, expands, and ceases producing endogenous urothelial cell types (K5-basal cells and I-cells), instead  K14-basal progenitors produce squamous cell daughters that express *Krt6a and Krt16*, which are not detected in the healthy urothelium.

The activation process in the skin is initiated by secretion of IL-1 and TNFa^84,85^, and is thought to be sustained by TNFa and TGF signaling, leading to activation of the NF-kB-signaling pathway and the associated inflammatory responses^86–89^. Consistent with this, we also observed upregulation of Il-1, TNF, IFNg, and NF-kB signaling in the bladder after short-term BBN treatment. Inflammatory reactions have long been implicated as a precursor prior to the formation of tumors in several types of cancer^90^, which in this case may be a response to neoantigen production. The basal cell activation program and squamous differentiation observed in response to injury in the urothelium and other epithelial barriers may have evolved as a mechanism for temporary repair; our studies suggest that this injury response may also be a hallmark of tumor formation in urothelial carcinoma.

RNA-seq analysis reveals downregulation of a large number of immune mediators in luminal tumors in *K5VP16;Pparg* mutants that based on the distribution of immune cells, are immune cold. In some types of cancer, with immune suppression, immune cells are observed near tumors but fail to penetrate. In luminal tumors of *K5VP16;Pparg* mutants, however, immunostaining reveals few if any immune cells in or near tumors, whereas control basal subtype tumors are immune infiltrated. Nf-kb is a master regulator of the immune response, and the *Rela* subunit p65, is transcriptionally regulated by *Pparg*^75,91^. We find abundant nuclear p65 in basal subtype tumors in controls lacking *VP16;Pparg* indicating active Nf-kb signaling, but p65 is barely detectable in luminal tumors of *K5VP16;Pparg* mutants. Although other possibilities exist, these observations raise the interesting possibility that *Pparg*-dependent suppression of Nf-kb activity may contribute to the immune cold phenotype in luminal tumors.

Intra-tumor heterogeneity and evolution have been observed in bladder cancer studies of tumor-derived organoids and mutational analysis of human tumors^52,92,93^. We observe altered histology at the base of luminal tumors in *K5VP16;Pparg* mutants that appears 4 months after Tamoxifen induction, but not before, suggesting that this phenotype is acquired over time. These domains express basal markers including Krt6a, and downregulate luminal markers. It is likely that new mutation acquisition from sustained exposure to BBN after tamoxifen induced expression of the *VP16;Pparg* transgene may be a driver of these changes, as bladder cancer has one of the highest tumor mutational burden. However, the observation that they form, and are in a basal position suggests that they may not be observed in biopsies of luminal subtype tumors taken from patients, or may remain after TURBT treatment, thus contributing to recurrence and progression from NMIBC to MIBC.

## Methods

### Mice

To generate an inducible *VP16;Pparg* mouse line, a *VP16;Pparg* cDNA was cloned into the AscI site of the CTV vector (Addgene #15912) via Gibson assembly method to generate pCTV-VP16PPARG gene-targeting vector placing a CAG promoter, a STOP cassette (Loxp-Neo-Loxp), the VP16PPARG cDNA and an IRES-EGFP into intron 1 of the ROSA26 gene. The VP16PPARG is expressed from a synthetic CAG promoter after the removal of the STOP cassette by Cre recombinase. The pCTV-VP16PPARG gene-targeting vector was linearized by AloI and electroporated into KV1 (129B6 hybrid) ES cells to generate targeted ES cells with modified ROSA26-CAG-STOP-VP16Pparg allele. The targeted ES cells were injected into the C57BL/6N blastocysts to generate germline chimeras. The male chimeras were bred to wild-type C57BL/6N females to transmit the ROSA26-CAG-STOP-VP16PPARG-IRES-EGFP allele. For conditional tissue-specific expression of the *VP16;Pparg*, the master ROSA26-CAG-STOP-VP16PPARG-IRES-EGFP mouse line was crossed to *Krt5Cre*^*ERT2*^ and induced by Tamoxifen. *mTmGfl/fl* (Gt(ROSA)26Sortm4(ACTB-tdTomato,-EGFP)Luo/J) mice were obtained from Jackson Laboratory (stock #007576). *K5Cre*^*ERT2*^ mice (FVB.Cg-Tg(KRT5cre/ERT2)2Ipc/JeldJ) were obtained from D. Metzger and P. Chambon. Primers used for genotyping are detailed in Supplementary Table 1. All work with mice was approved by and performed under the regulations of the Columbia University Institutional Animal Care and Use Committee. Animals were housed in the animal facility of Irving Cancer Research Center, Columbia University. Animals were housed in a standard cage of 75 square inches at or below the maximum cage density permitted by IACUC protocol. The temperature was maintained between 68 and 79 °F. Humidity was maintained between 30 and 70%. A timed-controlled lighting system was used for a uniform diurnal lighting cycle.

### Human specimens

Bladder tumors were obtained from patients undergoing TURBT at Columbia University Irving Medical Center. All patients gave informed consent under the Columbia University Institutional Review Board-approved protocols.

### Intraperitoneal tamoxifen induction

Mice (8–12 weeks of age) were injected with tamoxifen (Sigma cat#T5648) dissolved in corn oil, at a dose of 5 mg per 30 g body weight for three times over the course of one week.

### Ultrasound-guided tamoxifen induction

Mice (8–12 weeks of age) were injected with 150 μL of 4-OHT dissolved in DMEMF12/Tween 80 at a dose of 80 μg/mL every other day for two dosages using a VEVO 3100 Ultrasound Imaging System (FUJIFILM VisualSonics, Toronto, Canada) located within the mouse barrier in the Herbert Irving Cancer Center Small Animal Imaging facility.

### BBN treatment

BBN (0.05%; Sigma cat#B8061-1G) was administered in the water supply daily for 3.5–24 weeks to induce bladder cancer. Mice were euthanized at 3.5 or 24 weeks. All bladders were removed and embedded for sectioning and staining.

### Laser capture microdissection (LCM)

Five-month BBN *K5VP16;Pparg* and control bladders were flash-frozen sagitally in OCT and stored at −80°C. The specimen blocks were placed in the Leica CM3050 cryostat chamber for 5–10 min to temperature equilibrate. The tissue was sectioned at 5 μm on Arcturus PEN Membrane glass slides (Applied Biosystems cat#LCM0522) and the slides were immediately stored at −80°C. In all, 1 h prior to LCM, slides were removed from the −80°C and stained with Arcturus HistoGene Staining Solution (ThermoFisher Scientific cat#KIT0415) according to the manufacturer’s protocol. Tissue was then laser captured on a Zeiss AxioObserver.Z1 inverted microscope into AdhesiveCap 200 opaque caps (Zeiss cat#000830-19). In total, 240 μL of RLT Lysis Buffer (Qiagen cat#1015750) was then added to the captured tissue. The suspension was then processed for total RNA extraction. Samples with a RIN (regulation identification number) >6.5 were used for RNA-seq. These samples then were sequenced according to the steps listed in the RNA-Sequencing method.

### Single-cell dissociation

Cold active protease (CAP), was prepared and stored on ice (5 mM CaCl2, Sigma cat#21115, 10 mg/mL Bacillus Licheniformis protease, Sigma cat#P5459, 12.5 U/mL DNAse, Sigma cat#4716728001). Mice were perfused with 20 mL of CAP using a small vein infusion set (Kawasumi cat#D3K2-23G) and two 10 mL syringes per mouse. Bladders were dissected and immediately put in a 60 mm × 15 mm petri dish (Fisherbrand cat#FB0875713A) containing CAP on ice for 10 min. The bladders were then transferred to HBSS media (ThermoFisher cat#14170-112) containing 1% bovine serum albumin (Sigma cat#A2058) and 0.1% glucose, where they were inverted and the urothelium was manually separated from the stroma. The cell suspension was then collected into a 1.5 mL Eppendorf tube on ice and gently triturated until the cells were in a single-cell suspension.

### RNA-sequencing

For homeostasis experiments, *K5VP16;Pparg;mTmG* and *K5;mTmG* single-cell suspensions were filtered through a 70 μm filter (Fisherbrand cat#22363548) and then sorted on a BD Aria II Cell Sorter using a 130 μm nozzle aperture and 13 psi pressure to collect GFP-positive cells. Gating strategy was performed on BD FACSDiva Software v 8.0. Cells were then centrifuged at 500 × *g* for 30 min at 4°C. The supernatant was discarded, and the pellet was processed for total RNA extraction. Samples with a RIN (regulation identification number) >8 were used for RNA-seq. The libraries were prepared using the SMART-Seq® v4 Ultra® Low Input RNA Kit for Sequencing (TaKaRa) followed by Nextera XT (Illumina), both according to manufacturer’s instructions. They were sequenced to a targeted depth of 40 M 2 × 100 bp reads on a NovaSeq 6000 (Illumina). Differential expression analysis was performed by reading kallisto counts files into R using the R packages tximport (v.1.10.1) and biomaRt (v.2.34.2), and running DESeq2 (v.1.18) to generate log fold change values and *p* values between the two experimental groups. The heatmap and PCA plots were visualized after transforming the counts using VST (variance stabilizing transformation). Gene set analysis by ConsensusPathDB (Kamburov, A. et al. 2013) was used to identify significantly changed pathways.

### Heatmaps

RNA reads were aligned to the mouse reference genome(mm10) using STAR (v2.5.3a). The transcript levels were then quantified using SALMON (v0.9.1). Count data were extracted from SALMON output using Tximport (Bioconductor), and normalized and log2-transformed using DESeq2 (Bioconductor). Heatmaps were generated by ComplexHeatmaps^94^. Box plots of selected genes were generated by ggplot2(R 3.6.2). Significance was calculated using Mann–Whitney *U* test. Heatmaps used unsupervised clustering of *VP16;Pparg* mouse tumors and BBN/UPPL mouse tumors across previously established immune gene signatures^35^. Each gene signature was calculated as the *z* score of the average value of all genes included in the signature. Gene expression of murine tumors and human tumors from The Cancer Genome Atlas (TCGA; *n* = 408) was combined using upper quantile normalization and co-clustered using basal/luminal marker genes.

### Statistics and reproducibility

All quantitation was performed on at least three independent biological samples, using the ImageJ software. Data presented in box plots are mean values ± s.e.m. Statistical analysis was performed using the R version 4.0.4. In two group comparisons, statistical significance was determined using Mann–Whitney *U* test, considering a value of *p* < 0.05 as significant. The number of samples used in the experiments is included in figure legends. All immunostainings and H&E experiments were performed with at least three biological replicates and three technical replicates for each condition.

### Immunostaining

Bladders were embedded in paraffin and serial sections were generated. For immunohistochemistry, paraffin sections were deparaffinized using HistoClear and rehydrated through a series of Ethanol and 1× phosphate-buffered saline (PBS) washes. Antigen retrieval was performed by boiling slides for 15 min in pH 9 buffer or 30 min in pH 6 buffer. Primary antibodies in 1% horse serum were incubated overnight at 4°C. The next day, slides were washed with PBST three times for 10 min each, and secondary antibodies were applied for 90 min at room temperature. DAPI (4′,6-diamidino-2-phenylindole) was either applied as part of the secondary antibodies cocktail or for 10 min, for nuclear staining and then the slides were sealed with coverslips. The conditions of antibodies used are detailed in Supplementary Table 1.

### Fluorescent microscopy

Zeiss Axiovert 200 M microscope with Zeiss Apotome were used to collect immunofluorescent images. Bright-field images were collected using a Nikon Eclipse TE200 microscope. Data were analyzed using the Fiji package of ImageJ (v.1.0) and Photoshop screen overlay method (v. 21.1.0).

### Cartoon schematics

Schematics were adapted from “Oral Tolerance Experiment”, by BioRender.com (2021). Retrieved from https://app.biorender.com/biorender-templates.

### Reporting summary

Further information on research design is available in the Nature Research Reporting Summary linked to this article.

## Supplementary information

Supplementary Information

Reporting Summary

## Data Availability

The data sets generated during and/or analyzed during the current study are available in the Gene Expression Omnibus (GEO) repository under GEO accession number GSE172656. The TCGA bladder cancer data set can be accessed from The Cancer Genome Atlas at https://www.cancer.gov/about-nci/organization/ccg/research/structural-genomics/tcga. The remaining data are available within the Article and Supplementary Information Source data are provided with this paper.

## Source data

Source Data
