# Supplementary material for: Pparg signaling controls bladder cancer subtype and immune exclusion
Source: Nat Commun. 2021 Oct 25;12:6160. doi: 10.1038/s41467-021-26421-6 (PMC8545976; doi:10.1038/s41467-021-26421-6)
Supplement: Supplementary file 1 — Supplementary Information [file 41467_2021_26421_MOESM1_ESM.pdf]

Pparg signalling controls bladder cancer subtype and drives immune exclusion

Tate, et al.

Supplementary Fig. 1

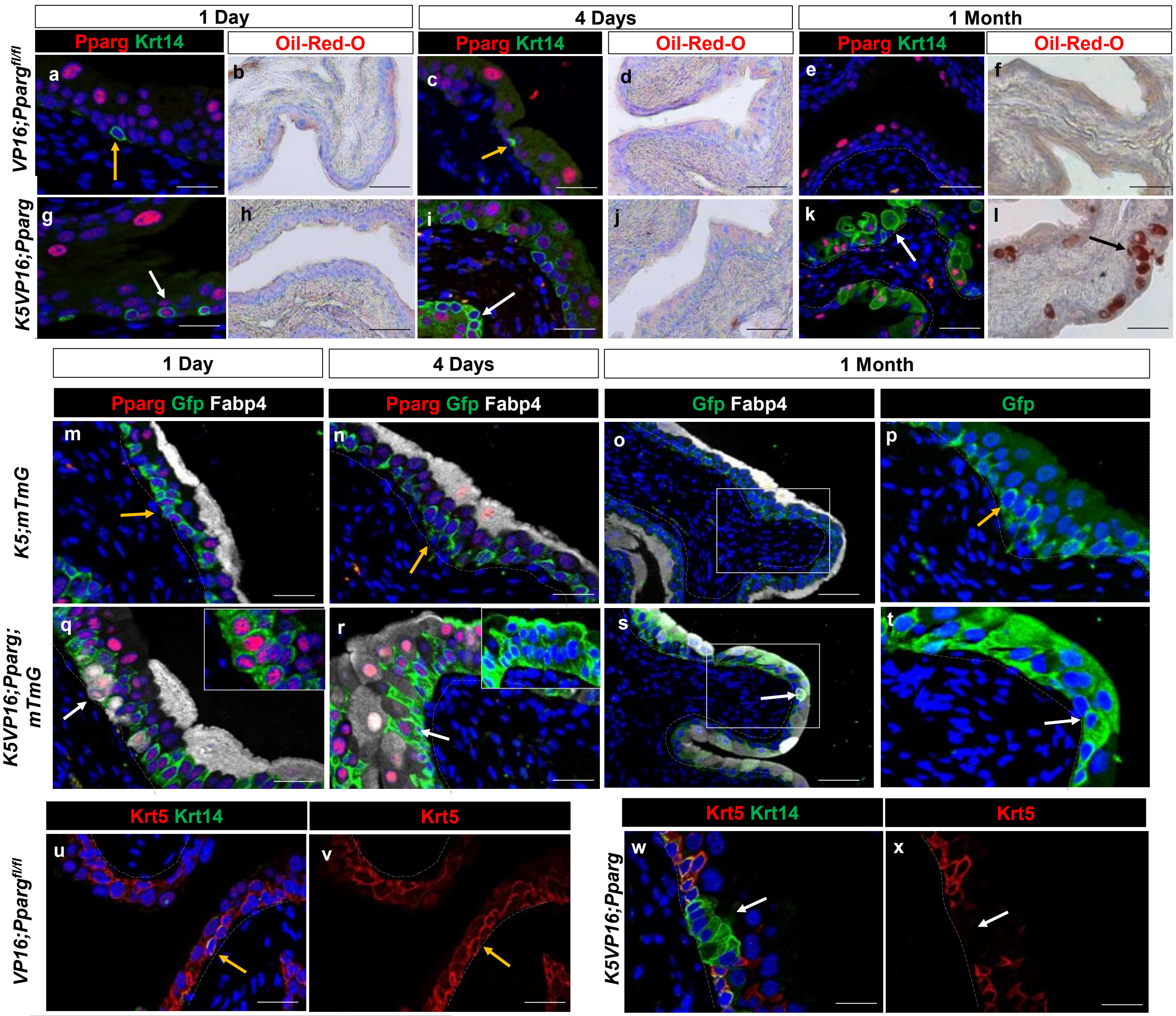

y

	Gene	FC	Pvalue
Luminal Markers	Upk1a	1.273411	0.101058
	Upk1b	1.771737	0.004768
	Upk2	1.831418	0.001319
	Upk3b	1.472945	0.021860
	Krt19	1.329701	0.041765
	Krt20	3.083968	2.04E-07
	Krt8	1.726677	0.000156
	Xbp1	1.424798	0.012264
	Cd24	1.618554	0.011801
	Snx31	1.57461	0.01846
	Fabp4	17.43987	2.90E-35
	Cldn8	1.370199	0.010296
Basal Markers	Krt5	0.829999	0.208742
	Col17a	0.303966	1.64E-10
	Trp63	0.579855	9.94E-07

z

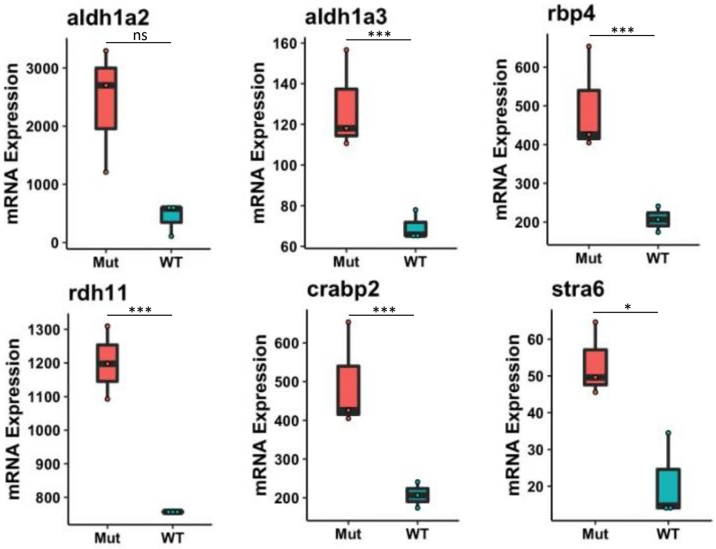

Supplementary Fig. 1. **(a-l)** Lipid droplet formation one month after Tamoxifen induction in *VP16;Pparg*$^{fl/fl}$ mutants. Pparg and Krt14 expression 1 day after Tamoxifen induction in controls **(a)** and in *K5VP16;Pparg* mutants **(g)**. Oil-Red-O staining in controls **(b)** and in *K5VP16;Pparg* mutants **(h)** 1 day after Tamoxifen induction. Expression of Pparg and Krt14 in controls **(c)** and in *K5VP16;Pparg* mutants **(i)** 4 days after Tamoxifen induction. Oil-Red-O staining in controls **(d)** and in *K5VP16;Pparg* mutants **(j)** 4 days after Tamoxifen induction. Pparg and Krt14 expression in controls **(e)** and in *K5VP16;Pparg* mutants **(k)** 1 month after Tamoxifen induction. Oil-Red-O staining of controls **(f)** and mutants **(l)** 1 month after induction. Expression of Pparg, Gfp, and Fabp4 1 day after Tamoxifen induction in *K5;mTmG* controls **(m)** and in *K5VP16;Pparg;mTmG* mutants **(q)**. Expression of Pparg, Gfp, and Fabp4 4 days after Tamoxifen induction in *K5;mTmG* controls **(n)** and in *K5VP16;Pparg;mTmG* mutants **(r)**. Expression of Gfp and Fabp4 1 month after Tamoxifen induction in *K5;mTmG* controls **(o)** and *K5VP16;Pparg;mTmG* mutants (**s**). Higher magnification image showing expression of Gfp 1 month after Tamoxifen induction in *K5;mTmG* controls **(p)** and *K5VP16;Pparg;mTmG* mutants **(t)**. Yellow arrows denote Gfp-positive control basal cells. White arrows denote Gfp-positive mutant cells expressing the transgene. Expression of Krt5 and Krt14 4 days after Tamoxifen induction in *VP16;Pparg*$^{fl/fl}$ controls **(u)** and *K5VP16;Pparg* mutants **(w)**. Expression of Krt5 4 days after Tamoxifen induction in controls **(v)** and in *K5VP16;Pparg* mutants **(x)** Yellow arrows denote control basal cells. White arrows denote mutant basal cells losing Krt5 expression. **(y)** Table showing the luminal/basal gene signature in based on RNA-seq analysis of urothelium from *K5;mTmG* controls and *K5VP16;Pparg* mutants 4 days post-Tamoxifen induction. **(z)** mRNA expression showing increased expression of genes involved in RA signaling in*K5VP16;Pparg;mTmG* mutants (n=3) compared to controls (n=3) 4 days after Tamoxifen induction. Box plots display minima, maxima, and interquartile range (IQR). Significance was calculated by one-sided Mann-Whitney U test (aldh1a2 p=0.67, aldh1a3 p=0.0059, rbp4 p=0.00061, rdh11 p=0.00032, crabp2 p=0.00032, stra6 p=0.036). ns=not significant; *p≤0.05; **0.05 ≤p≤0.01; ***0.01 ≤p≤0.0001. Scale bars, 50 μm.

Supplementary Fig. 2

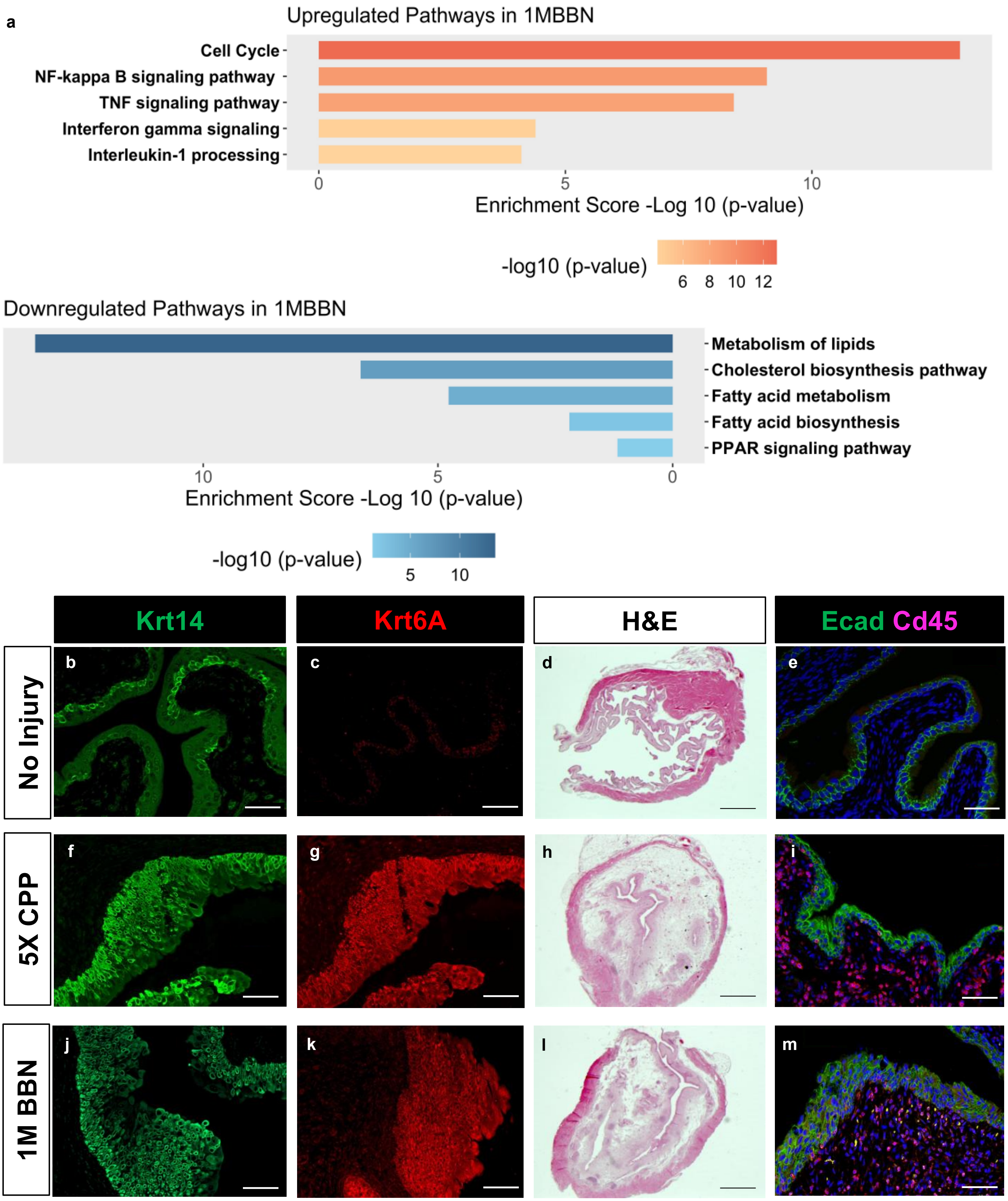

Supplementary Fig. 2. (a) Up-regulated and down-regulated pathways from RNA-seq analysis of controls and bladders after 1MBBN treatment. p-values were calculated by hypergeometric test and corrected for multiple testing. **(b-m)** Repeated CPP treatment induces a similar activation program in the urothelium as that induced by 1M of BBN treatment. **(b,f,j)** Expression of Krt14 in controls **(b)**, animals treated with 5X CPP **(f)** and animals treated with BBN for 1 month **(j)**. **(c,g,k)** Expression of Krt6A in controls **(c)**, animals treated with 5X CPP **(g)**, and animals treated with BBN for 1 month **(k)**. H&E staining of bladders from controls **(d)**, from animals treated with 5X CPP **(h)**, and from animals treated with BBN for 1 month **(l)**. **(e,i,m)** E-cadherin and Cd45 expression in controls **(e)**, in animals treated with 5X CPP **(i)** and in animals after 1 month of BBN treatment **(m)**. Scale bars in **(b,c,e,f,g,I,j,k,m)** 50 μm. Scale bars in **(d,h,l)** 200 μm.

Supplementary Figure 3

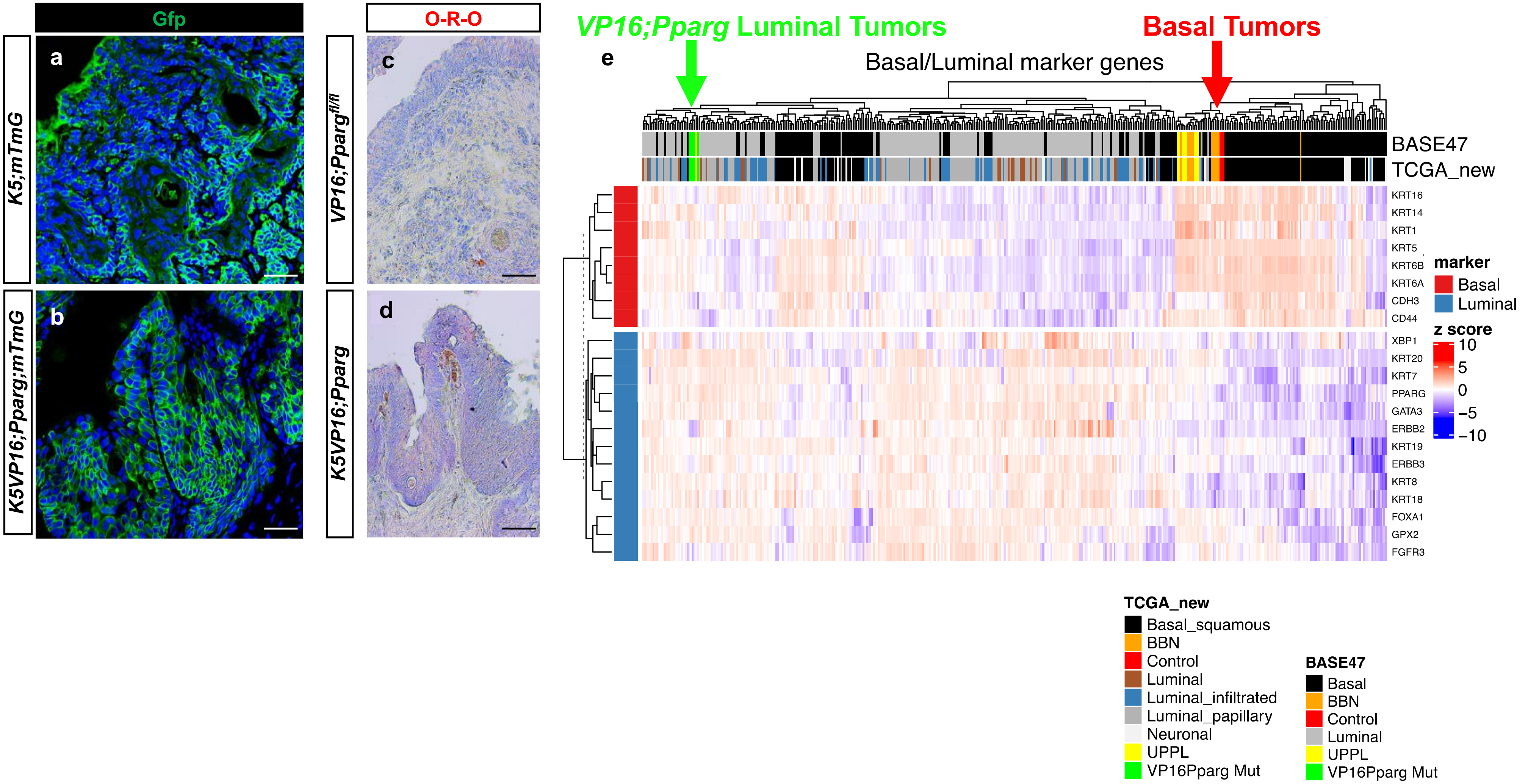

Supplementary Fig. 3. (a,b) Lineage tracing showing expression of Gfp-labeled basal cells and their daughters in a *K5;mTmG* control tumor **(a)** and in a *K5VP16;Pparg;mTmG* mutant tumor **(b)**. Oil-Red-O staining of a *VP16;Pparg*$^{fl/fl}$ control tumor **(c)** and *K5VP16;Pparg* mutant tumor **(d)** 4 months after Tamoxifen induction. **(e)** Heatmap showing co-clustering of tumors from *K5;mTmG* controls and *K5VP16;Pparg* mutant tumors with luminal and basal tumors respectively. Data includes the TCGA BLCA data set, BASE47 base classifier data set, BBN treated tumors and UPPL tumors. Scale bars 50μm.

Supplementary Fig. 4

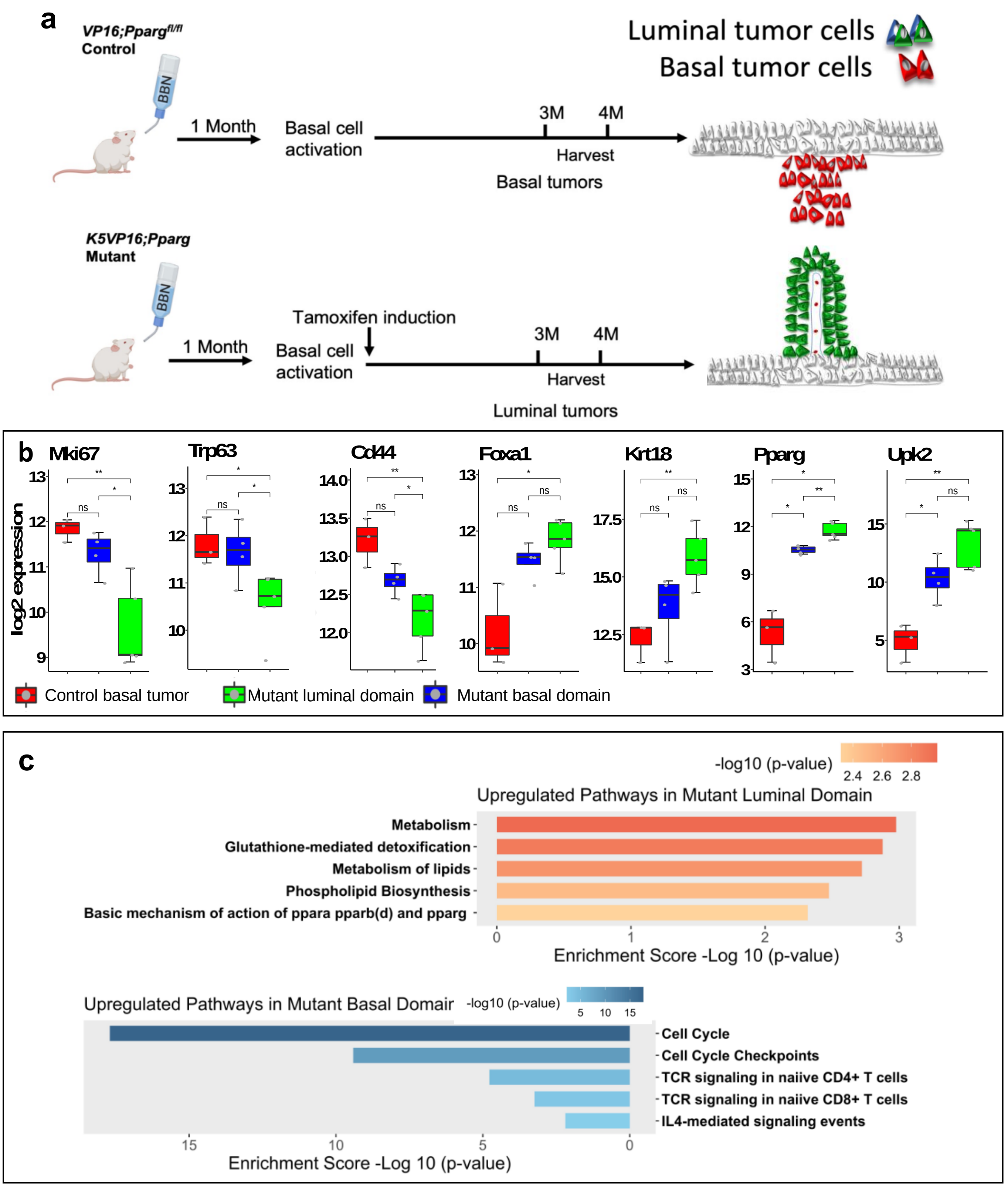

Supplementary Fig. 4. (a) Schematic of luminal and basal tumor formation in *K5;mTmG* controls and *K5VP16;Pparg;mTmG* mutants, respectively. **(b)** Expression levels mRNA encoding *Mki67, Trp63, Cd44, Foxa1, Krt18, Pparg,* and *Upk2* from *K5;mTmG* in basal control tumors (n=3) compared to luminal (n=4) and basal domains (n=4) of *K5VP16;Pparg;mTmG* mutant tumors at 4 months. Box plots display minima, maxima, and interquartile range (IQR). Significance was calculated by Mann-Whitney U test. ns=not significant; *p≤0.05; **0.05 ≤p≤0.01. **(c)** Upregulated and downregulated signaling pathways based on RNA-seq analysis of *K5VP16;Pparg* mutant luminal domain compared to the *K5VP16;Pparg* mutant basal domain at 4 months. p-values were calculated by hypergeometric test and corrected for multiple testing.

Supplementary Fig. 5

a

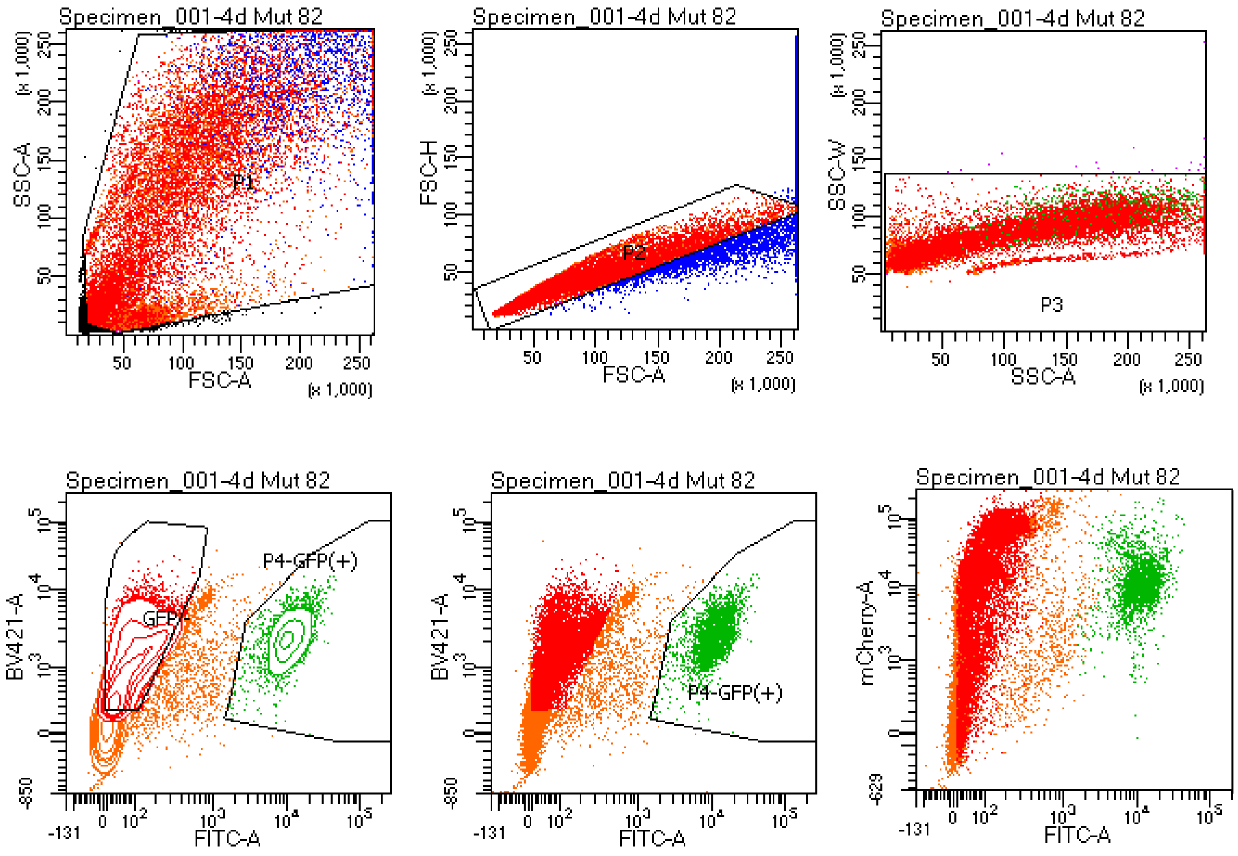

Tube: 4d Mut 82

Population	#Events	%Parent	%Total
All Events	30,000	####	100.0
P1	25,796	86.0	86.0
P2	18,504	71.7	61.7
P3	18,469	99.8	61.6
P4-GFP(+)	2,664	14.4	8.9
GFP -	8,804	47.7	29.3

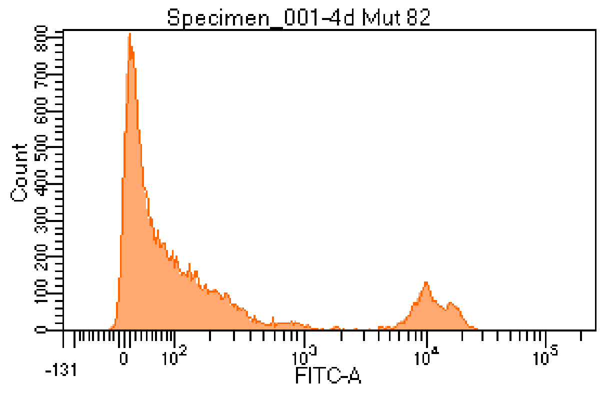

Supplementary Fig. 5. (a) Gating strategy used to collect GFP+ cells in homeostatic RNAseq.

Supplementary Table 1. Key resources used in the study

ANTIBODIES	SOURCE	IDENTIFIER	CLONE	DILUTION
Chicken Polyclonal Anti-Keratin 14	Biolegend	Cat#906001	N/A	1:400
Chicken Polyclonal Anti-Keratin 5	Biolegend	Cat#905901	N/A	1:400
Rabbit Polyclonal Anti-Krt6A	LSBio	Cat#LS-B12036-100	N/A	1:2000
Mouse Monoclonal Anti-Cytokeratin 20	AgilentDako	Cat# M701929-2		1:200
Rabbit Polyclonal Anti-PPARG	Cell Signaling Technology	Cat# 2435	N/A	1:200
Goat Polyclonal Anti-FABP4	R&D Systems	Cat# AF1443	N/A	1:1000
Mouse Monoclonal Anti-FOXA1	Seven Hills Bioreagents	Cat# WMAB-2F83	2F83	1:1000
Mouse Monoclonal Anti-p21/CDKN1A/WAF1	LSBio	Cat# LS-C389956	HJ21	1:200
Rabbit Polyclonal Anti-p63	GeneTex	Cat#GTX102425	N/A	1:300
Goat Polyclonal Anti-p63	R&D Systems	Cat#AF1916	N/A	1:200
Rat Monoclonal Anti-CD45	BD Bioscience	Cat#550539	30F11	1:100
Rabbit Polyclonal Anti-Cytokeratin 18	Abcam	Cat#ab52948	N/A	1:500
Rabbit Polyclonal Anti-Ki67	Abcam	Cat#ab15580	N/A	1:200
Chicken Polyclonal Anti-GFP	Aves Labs	Cat#GFP1020	N/A	1:300
Goat Polyclonal Anti-E-Cadherin	R&D Systems	Cat#AF748	N/A	1:400

Rabbit Polyclonal NFKB p65	Abcam	Cat#AB19870	N/A	1:300
Rabbit Polyclonal Laminin	Sigma	Cat#L9393	N/A	1:100
Mouse Monoclonal SMA-CY3	Sigma	Cat#C6198	1A4	1:500
Alexa Fluor 488 Donkey Anti-Rabbit IgG	Jackson Immunoresearch	Cat#711-545-152	N/A	1:700
Alexa Fluor 488 Donkey Anti-Mouse IgG	Jackson Immunoresearch	Cat#711-545-150	N/A	1:700
Alexa Fluor 488 Donkey Anti-Chicken IgG	Jackson Immunoresearch	Cat#703-545-155	N/A	1:700
Alexa Fluor 488 Donkey Anti-Goat IgG	Jackson Immunoresearch	Cat#705-545-003	N/A	1:700
Cy3 Donkey Anti-Rabbit IgG	Jackson Immunoresearch	Cat#711-165-152	N/A	1:700
Alexa Fluor 594 Donkey Anti-Mouse IgG	Jackson Immunoresearch	Cat#715-585-151	N/A	1:700
Alexa Fluor 594 Donkey Anti-Chicken IgG	Jackson Immunoresearch	Cat#703-585-155	N/A	1:700
Alexa Fluor 594 Donkey Anti-Goat IgG	Jackson Immunoresearch	Cat#705-585-147	N/A	1:700
Alexa Fluor 647 Donkey Anti-MouseIgG	Jackson Immunoresearch	Cat#715-605-150	N/A	1:400
Alexa Fluor 647 Donkey Anti-Rabbit IgG	Jackson Immunoresearch	Cat#711-605-152	N/A	1:400
Alexa Fluor 647 Donkey Anti-Chicken IgG	Jackson Immunoresearch	Cat#703-605-155	N/A	1:400
Alexa Fluor 647 Donkey Anti-Goat IgG	Jackson Immunoresearch	Cat#705-605-003	N/A	1:400

**REAGENT or RESOURCE**	**SOURCE**	**IDENTIFIER**
**Biological samples**		
Human bladder tumors samples	Columbia University Irving Medical Center	N/A

**Chemicals, Peptides, and Recombinant proteins**		
N-butyl-N-(4-hydroxybutl) nitrosamine	Sigma	Cat#B8061-1G
Hank's Balanced Salt Solution (HBSS)	ThermoFisher Scientific	Cat#14170-112
Bovine Serum Albumin	Sigma	Cat#A2058
Bacillus licheniformis protease	Sigma	Cat#P5459
$CaCl_2$	Sigma	Cat#21115
DNAse I recombinant	Sigma	Cat#4716728001
RLT lysis buffer	Qiagen	Cat#1015750
Antigen unmasking solution	Vector Labs	Cat#H3300
Citrate buffer	Thermo Fisher Scientific	Cat#AP-9003-500
Horse Serum, heat inactivated	Gibco	Cat#26050070
Tween80	Sigma	Cat#P1754
DMEM/F12	Thermo Fisher Scientific	Cat#H7904
4-Hydroxytamoxifen	Sigma	Cat#11320033
Tamoxifen	Sigma	Cat#T5648
Histogene Staining Solution	TherFisher Scientific	Cat#KIT0425
**Critical commercial assays**		
SMART-Seq v4 Ultra Low Input RNA Kit for Sequencing	TaKaRa	Cat#634889
**Deposited data**		

Raw RNA-seq files	This paper	GEO: GSE172656
**Experimental Models: Organisms/Strains**		
Mouse: *VP16;Pparg*	This paper	
Mouse: *mTmGfl/fl* (Gt(ROSA)26Sortm4(ACTB-tdTomato,-EGFP)Luo/J)	Jackson Laboratory	Cat#007576
Mouse: FVB.Cg-Tg(KRT5-cre/ERT2)2Ipc/JeldJ (K5Cre^{ERT2})	D. Metzger<br>P. Chambon	N/A
**Oligonucleotides**		
Primer: *VP16;Pparg*<br>Mutant forward: 5'-CTGCATTCTAGTTGTGGTTTGTCCA-3'<br>Mutant reverse:5'-ATCGGTAAACATCTGCTCAAACTCG-3'<br>Wild type forward 5'-CCCAAAGTCGCTCTGAGTTGTTATC-3'<br>Wild type reverse 5'-AACTCGGGTGAGCATGTCTTTAATC-3'	This paper	N/A
Primer: *Krt5Cre^{ERT2}*<br>Forward: 5′-ATTTGCCTGCATTACCGGTC-3′<br>Reverse: 5′-ATCAACGTTTTGTTTTCGGA-3′	Indira et al, 1999	NA
Primer: *mTmG*	Muzumdar et al, 2007	NA

Common 5′-CTCTGCTGCCTCCTGGCTTCT-3′<br>Mutant 5′-TCAATGGGCGGGGGTCGTT-3′<br>Wild type 5′-CGAGGCGGATCACAAGCAATA-3′		
**Software and Algorithms**		
R version 4.0.4	R Core Team, 2016	http://www.r-project.org/
DESeq2	Love et al., 2014	https://github.com/mikelove/<br>DEseq2
Gene Set Enrichment Analysis, v4.1.0	Subramanian et al., 2005	https://www.gsea-<br>msigdb.org/gsea/index.jsp
**Other**		
VEVO 3100Ultasound Imaging System	FUJIFILM VisualSonics	N/A
Zeiss AxioObserver.Z1 inverted microscope for laser capture microdissection	Zeiss	N/A
