# Supplementary material for: Pparg signaling controls bladder cancer subtype and immune exclusion
Source: Nat Commun. 2021 Oct 25;12:6160. doi: 10.1038/s41467-021-26421-6 (PMC8545976; doi:10.1038/s41467-021-26421-6)
Supplement: Supplementary file 2 — Reporting Summary [file 41467_2021_26421_MOESM2_ESM.pdf]

nature portfolio

Corresponding author(s): Cathy Mendelsohn

Last updated by author(s): Aug 20, 2021

Reporting Summary

Nature Portfolio wishes to improve the reproducibility of the work that we publish. This form provides structure for consistency and transparency in reporting. For further information on Nature Portfolio policies, see our Editorial Policies and the Editorial Policy Checklist.

Statistics

For all statistical analyses, confirm that the following items are present in the figure legend, table legend, main text, or Methods section.

n/a	Confirmed	
☐	☒	The exact sample size (n) for each experimental group/condition, given as a discrete number and unit of measurement
☐	☒	A statement on whether measurements were taken from distinct samples or whether the same sample was measured repeatedly
☐	☒	The statistical test(s) used AND whether they are one- or two-sided<br>*Only common tests should be described solely by name; describe more complex techniques in the Methods section.*
☐	☒	A description of all covariates tested
☐	☒	A description of any assumptions or corrections, such as tests of normality and adjustment for multiple comparisons
☒	☐	A full description of the statistical parameters including central tendency (e.g. means) or other basic estimates (e.g. regression coefficient) AND variation (e.g. standard deviation) or associated estimates of uncertainty (e.g. confidence intervals)
☐	☒	For null hypothesis testing, the test statistic (e.g. F, t, r) with confidence intervals, effect sizes, degrees of freedom and P value noted<br>*Give P values as exact values whenever suitable.*
☒	☐	For Bayesian analysis, information on the choice of priors and Markov chain Monte Carlo settings
☒	☐	For hierarchical and complex designs, identification of the appropriate level for tests and full reporting of outcomes
☒	☐	Estimates of effect sizes (e.g. Cohen's d, Pearson's r), indicating how they were calculated

Our web collection on statistics for biologists contains articles on many of the points above.

Software and code

Policy information about availability of computer code

Data collection	Zeiss AxioObserver.Z1
Data analysis	DEseq2 (v.1.18) , Gene Set Enrichment Analysis, v4.1.0, tximport (v.1.10.1), biomaRt (v.2.34.2), STAR (v2.5.3a), ImageJ2/FIJI (v.1.0), Photoshop (21.1.0) ('screen') overlay method, SALMON (v0.9.1), BD FACSDiva Software v 8.0

For manuscripts utilizing custom algorithms or software that are central to the research but not yet described in published literature, software must be made available to editors and reviewers. We strongly encourage code deposition in a community repository (e.g. GitHub). See the Nature Portfolio guidelines for submitting code & software for further information.

Data

Policy information about availability of data

All manuscripts must include a data availability statement. This statement should provide the following information, where applicable:

- Accession codes, unique identifiers, or web links for publicly available datasets
- A description of any restrictions on data availability
- For clinical datasets or third party data, please ensure that the statement adheres to our policy

The datasets generated during and/or analyzed during the current study are available in the GEO repository, GEO accession GSE172656

Field-specific reporting

Please select the one below that is the best fit for your research. If you are not sure, read the appropriate sections before making your selection.

- [x] Life sciences
- [ ] Behavioural & social sciences
- [ ] Ecological, evolutionary & environmental sciences

For a reference copy of the document with all sections, see nature.com/documents/nr-reporting-summary-flat.pdf

Life sciences study design

All studies must disclose on these points even when the disclosure is negative.

Sample size	For homeostatic experiments involving immunostaining, at least 3 biological replicates and 5 technical replicates per condition were used for each experiment. For homeostatic experiments involving RNAseq, at least 5 biological replicates were used per condition. We determined this to be sufficient due to low variability between samples during homeostasis and the yield of usable mice at the time of experiment. At least 5 biological replicates were used for tumor studies. We determined this higher sample size to be sufficient to distinguish phenotype between controls and mutants.<br>All sample sizes used in this study were in line with current field standard for mice experiments, based on the minimum number of mice required to detect significance with an alpha rate set at 0.05 in a standardly powered experiment.
Data exclusions	Outliers from RNAseq were identified based on PCA plots. Samples that did not closely cluster with its corresponding group were removed.
Replication	Each experiment was repeated at least three times with consistent results.
Randomization	Bladder sections were selected randomly for staining for homeostatic studies. Bladders were sectioned sagittally from end to end, resulting in approximately 10 series with 10 slides in each series. 3 slides from front- mid- and rear-parts of the bladder were selected randomly for each experiment. For tumor studies, bladder sections with the most prominent tumor structures were selected for controls and mutants based on H&E staining. For sequencing experiments, available age-matched mice were selected and allocated to control or experimental groups based on their genotype.
Blinding	Blinding was not possible as experimental conditions were evident from the image data.

Reporting for specific materials, systems and methods

We require information from authors about some types of materials, experimental systems and methods used in many studies. Here, indicate whether each material, system or method listed is relevant to your study. If you are not sure if a list item applies to your research, read the appropriate section before selecting a response.

Materials & experimental systems

n/a	Involved in the study
[ ]	[x] Antibodies
[x]	[ ] Eukaryotic cell lines
[x]	[ ] Palaeontology and archaeology
[ ]	[x] Animals and other organisms
[ ]	[x] Human research participants
[x]	[ ] Clinical data
[x]	[ ] Dual use research of concern

Methods

n/a	Involved in the study
[x]	[ ] ChIP-seq
[ ]	[x] Flow cytometry
[x]	[ ] MRI-based neuroimaging

Antibodies

Antibodies used

ANTIBODIES SOURCE IDENTIFIER CLONE DILUTION
Chicken Polyclonal Anti-Keratin 14 Biolegend Cat#906001 N/A 1:400
Chicken Polyclonal Anti-Keratin 5 Biolegend Cat#905901 N/A 1:400
Rabbit Polyclonal Anti-Krt6A LSBio Cat#LS-B12036-100 N/A 1:2000
Mouse Monoclonal Anti-Cytokeratin 20 AgilentDako Cat# M701929-2
1:200
Rabbit Polyclonal Anti-PPARG Cell Signaling Technology Cat# 2435 N/A 1:200
Goat Polyclonal Anti-FABP4 R&D Systems Cat# AF1443 N/A 1:1000
Mouse Monoclonal Anti-FOXA1 Seven Hills Bioreagents Cat# WMAB-2F83
2F83 1:1000
Mouse Monoclonal Anti-p21/CDKN1A/WAF1 LSBio Cat# LS-C389956 HJ21 1:200
Rabbit Polyclonal Anti-p63 GeneTex Cat#GTX102425 N/A 1:300
Goat Polyclonal Anti-p63 R&D Systems Cat#AF1916 N/A 1:200
Rat Monoclonal Anti-CD45 BD Bioscience Cat#550539 30F11 1:100

Rabbit Polyclonal Anti-Cytokeratin 18 Abcam Cat#ab52948 N/A 1:500
Rabbit Polyclonal Anti-Ki67 Abcam Cat#ab15580 N/A 1:200
Chicken Polyclonal Anti-GFP Aves Labs Cat#GFP1020 N/A 1:300
Goat Polyclonal Anti-E-Cadherin R&D Systems Cat#AF748 N/A 1:400
Rabbit Polyclonal NFKB p65 Abcam Cat#AB19870 N/A 1:300
Rabbit Polyclonal Laminin Sigma Cat#L9393 N/A 1:100
Mouse Monoclonal SMA-CY3 Sigma Cat#C6198 1A4 1:500
Alexa Fluor 488 Donkey Anti-Rabbit IgG Jackson Immunoresearch Cat#711-545-152 N/A 1:700
Alexa Fluor 488 Donkey Anti-Mouse IgG Jackson Immunoresearch Cat#711-545-150 N/A 1:700
Alexa Fluor 488 Donkey Anti-Chicken IgG Jackson Immunoresearch Cat#703-545-155 N/A 1:700
Alexa Fluor 488 Donkey Anti-Goat IgG Jackson Immunoresearch Cat#705-545-003 N/A 1:700
Cy3 Donkey Anti-Rabbit IgG Jackson Immunoresearch Cat#711-165-152 N/A 1:700
Alexa Fluor 594 Donkey Anti-Mouse IgG Jackson Immunoresearch Cat#715-585-151 N/A 1:700
Alexa Fluor 594 Donkey Anti-Chicken IgG Jackson Immunoresearch Cat#703-585-155 N/A 1:700
Alexa Fluor 594 Donkey Anti-Goat IgG Jackson Immunoresearch Cat#705-585-147 N/A 1:700
Alexa Fluor 647 Donkey Anti-MouseIgG Jackson Immunoresearch Cat#715-605-150 N/A 1:400
Alexa Fluor 647 Donkey Anti-Rabbit IgG Jackson Immunoresearch Cat#711-605-152 N/A 1:400
Alexa Fluor 647 Donkey Anti-Chicken IgG Jackson Immunoresearch Cat#703-605-155 N/A 1:400
Alexa Fluor 647 Donkey Anti-Goat IgG Jackson Immunoresearch Cat#705-605-003 N/A 1:400

Validation

All antibodies have been validated with immunostaining on positive and negative control tissues. Multiple dilution and antigen retrieval methods were tested. P63, Krt20, Ki67, E-cadherin, Krt14, Pparg, Krt5, Cd45, Krt6a, SMA, and Nfkb p65 have been validated in our previous publication (Liu and Tate et al., 2019).

Animals and other organisms

Policy information about studies involving animals; ARRIVE guidelines recommended for reporting animal research

Laboratory animals

Mixed background mice (C57BL/6J, Swiss Webster)(male and female) age between 8weeks-30weeks were used for bladder harvest. Animals were housed in standard cage of 75 square inches at or below maximum cage density permitted by IACUC protocol. Temperature was maintained between 68-79°F. Humidity was maintained between 30-70%. A timed-controlled lighting system was used for an uniform diurnal lighting cycle.

Wild animals

No wild animals were used in this study.

Field-collected samples

No field-collected samples were used in this study.

Ethics oversight

All work with mice was approved by and performed under the regulations of the Columbia University Institutional Animal Care and Use Committee.

Note that full information on the approval of the study protocol must also be provided in the manuscript.

Human research participants

Policy information about studies involving human research participants

Population characteristics

Patients undergoing surgery in the Department of Urology at Columbia University Irving Medical Center.

Recruitment

Patients diagnosed with bladder cancer and are undergoing for tumor resection were recruited.

Ethics oversight

Protocol AAAN8850 was approved by IRB committee

Note that full information on the approval of the study protocol must also be provided in the manuscript.

Flow Cytometry

Plots

Confirm that:

- [x] The axis labels state the marker and fluorochrome used (e.g. CD4-FITC).
- [x] The axis scales are clearly visible. Include numbers along axes only for bottom left plot of group (a 'group' is an analysis of identical markers).
- [x] All plots are contour plots with outliers or pseudocolor plots.
- [x] A numerical value for number of cells or percentage (with statistics) is provided.

Methodology

Sample preparation

Cold active protease (CAP), was prepared and stored on ice (5mM CaCl2, Sigma cat#21115, 10mg/mL Bacillus Licheniformis protease, Sigma cat#P5459, 12.5U/mL DNAse, Sigma cat#4716728001). Mice were perfused with 20 mL of CAP using a small

	vein infusion set (Kawasumi cat#D3K2-23G) and two 10 mL syringes per mouse. Bladders were dissected and immediately put in a 60mm x 15mm petri dish (Fisherbrand cat#FB0875713A) containing CAP on ice for 10 min. The bladders were then transferred to HBSS media (ThermoFisher cat#14170-112) containing 1% Bovine Serum Albumin (Sigma cat#A2058) and 0.1% glucose, where they were inverted and the urothelium was manually separated from the stroma. The cell suspension was then collected into a 1.5mL Eppendorf tube on ice and gently triturated until the cells were in a single cell suspension.
Instrument	BD Aria II Cell Sorter
Software	BD FACSDiva
Cell population abundance	4-Way-Purity Mode was used for purity. Target cell population made up ~10-20% of total cell population.
Gating strategy	Using the FSC/SSC gating, debris was removed by gating on the main cell population. Positivity threshold for each sample was defined on the basis of GFP- sample. Identical positivity threshold was applied to all samples within cell line.

☒ Tick this box to confirm that a figure exemplifying the gating strategy is provided in the Supplementary Information.
